# Room-Temperature Ferromagnetism in Epitaxial Bilayer FeSb/SrTiO_3_(001) Terminated with a Kagome Lattice

**DOI:** 10.1021/acs.nanolett.3c03415

**Published:** 2023-11-01

**Authors:** Huimin Zhang, Qinxi Liu, Liangzi Deng, Yanjun Ma, Samira Daneshmandi, Cheng Cen, Chenyu Zhang, Paul M. Voyles, Xue Jiang, Jijun Zhao, Ching-Wu Chu, Zheng Gai, Lian Li

**Affiliations:** ‡ Department of Physics and Astronomy, West Virginia University, Morgantown, West Virginia 26506, United States; § State Key Laboratory of Structural Analysis, Optimization and CAE Software for Industrial Equipment, Dalian University of Technology, Dalian, 116024, China; ⊥ Key Laboratory of Materials Modification by Laser, Ion and Electron Beams (Dalian University of Technology), Ministry of Education, Dalian 116024, China; ∥ Department of Physics and Texas Center for Superconductivity, University of Houston, Houston, Texas, 77204, United States; # Beijing National Laboratory for Condensed Matter Physics and Institute of Physics, Chinese Academy of Sciences, Beijing 100190, China; □ Department of Materials Science and Engineering, University of Wisconsin−Madison, Madison, Wisconsin 53706, United States; ○ Center for Nanophase Materials Sciences, Oak Ridge National Laboratory, Oak Ridge, Tennessee, 37831 United States

**Keywords:** room-temperature ferromagnetism, FeSb films, MBE, Kagome lattice

## Abstract

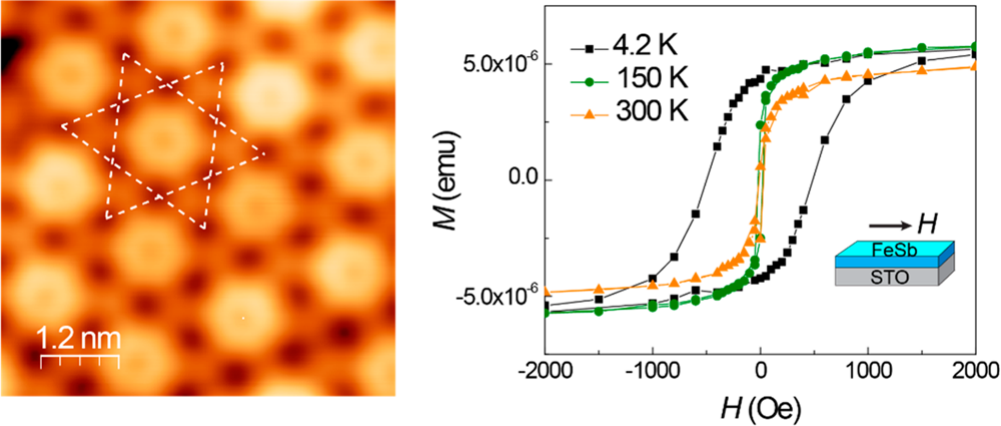

Two-dimensional (2D) magnets exhibit unique physical properties for potential applications in spintronics. To date, most 2D ferromagnets are obtained by mechanical exfoliation of bulk materials with van der Waals interlayer interactions, and the synthesis of single- or few-layer 2D ferromagnets with strong interlayer coupling remains experimentally challenging. Here, we report the epitaxial growth of 2D non-van der Waals ferromagnetic bilayer FeSb on SrTiO_3_(001) substrates stabilized by strong coupling to the substrate, which exhibits in-plane magnetic anisotropy and a Curie temperature above 390 K. *In situ* low-temperature scanning tunneling microscopy/spectroscopy and density-functional theory calculations further reveal that an Fe Kagome layer terminates the bilayer FeSb. Our results open a new avenue for further exploring emergent quantum phenomena from the interplay of ferromagnetism and topology for application in spintronics.

Ferromagnetic (FM) order in two-dimensional (2D) materials has attracted extensive attention owing to its tremendous application prospects in data storage,^1^ sensors,^2^ high-efficiency spin-based computers, and other nanoscale devices.^3,4^ Understanding emergent phenomena in 2D hybrid magnetic structures is crucial in laying a solid foundation for realizing these potential applications.^5,6^ To date, substantial theoretical^7^ and experimental^8^ efforts have been made to achieve long-range ferromagnetic order in 2D materials at room temperature (RT). Such order was initially predicted to be absent at finite temperatures (*T* > 0) for isotropic 2D systems based on the Mermin-Wagner theorem.^9^ However, magnetic anisotropy was later found to quench thermal fluctuations^10,11,1^ and help stabilize the long-range FM order in the 2D limit. Most recently discovered 2D magnets are based on layered materials,^11−17^ in which the layers are held together by van der Waals (vdW) interactions. Even though 2D magnetism is well established in the single-layer limit, the FM orders in vdW materials are generally fragile, with Curie temperature (*T*_c_) well below RT. For instance, the *T*_*c*_ in CrI_3_,^12^ CrCl_3_,^18^ CrBr_3_,^19^ Cr_2_Ge_2_Te_6_,^11^ and CrSBr^20^ single-layers are reported to be 45, 13, 27, 44, and 146 K, respectively. This has been attributed to enhanced spin fluctuations at reduced dimensions or relatively weak exchange interactions. In addition, 2D vdW materials are usually not air-stable, and the size of flakes exfoliated from bulk crystals is typically limited to micrometers, which is not ideal for practical applications.

One route to overcome these drawbacks is to develop 2D non-vdW magnetic materials^21^ that exhibit different magnetic properties than their bulk counterparts. For example, 2D hematene (α-Fe_2_O_3_) is a ferromagnet, while bulk hematite is antiferromagnetic.^22^ These materials have already shown unique advantages of easily modulated spin behavior and high Curie temperature,^21^ as observed in α-Fe_2_O_3_,^22^ ilmenene (FeTiO_3_),^23^ chromiteen (FeCr_2_O_4_)^24^), and transition-metal chalcogenides (TMDCs) including Cr_2_S_3_,^25^ CrSe,^26^ CrTe,^27^ Cr_2_Te_3_,^28^ MnTe,^29^ and *h*-FeTe.^30^

Another non-vdW material class is Kagome magnets, which exhibit emerging topological behavior with a complex magnetic phase diagram due to frustration and competing magnetic interactions.^31^ For example, FeSn, consisting of Sn honeycomb layers and corner-sharing Fe_3_Sn Kagome ferromagnetically coupled in-plane and antiferromagnetically out-of-plane, hosts linearly dispersing Dirac states and flat bands.^32^ Exploring these 2D non-vdW materials with long-range ferromagnetic order is an active area of research to probe the interplay of geometrical frustration, topology, and magnetism.

In this work, we synthesize bilayer FeSb films on SrTiO_3_(001) substrates by molecular beam epitaxy (MBE) and perform *in situ* low-temperature scanning tunneling microscopy/spectroscopy (LT-STM/S), *ex-situ* scanning transmission electron microscopy (STEM), magnetization measurements, and density-functional theory (DFT) calculations. The results show that while bulk FeSb is an antiferromagnet,^33^ the bilayer FeSb film is a non-vdW ferromagnet stabilized by strong coupling to the STO substrate, which exhibits a robust long-range FM order above 300 K with in-plane anisotropy. Furthermore, LT-STM/S measurements and DFT calculations show that the bilayer FeSb is terminated by a surface Fe Kagome lattice, offering a unique 2D system that explores emergent quantum phenomena from the interplay of ferromagnetism and topology.

## Epitaxial Growth of FeSb/SrTiO_3_(001) Films

**Figure 1:**
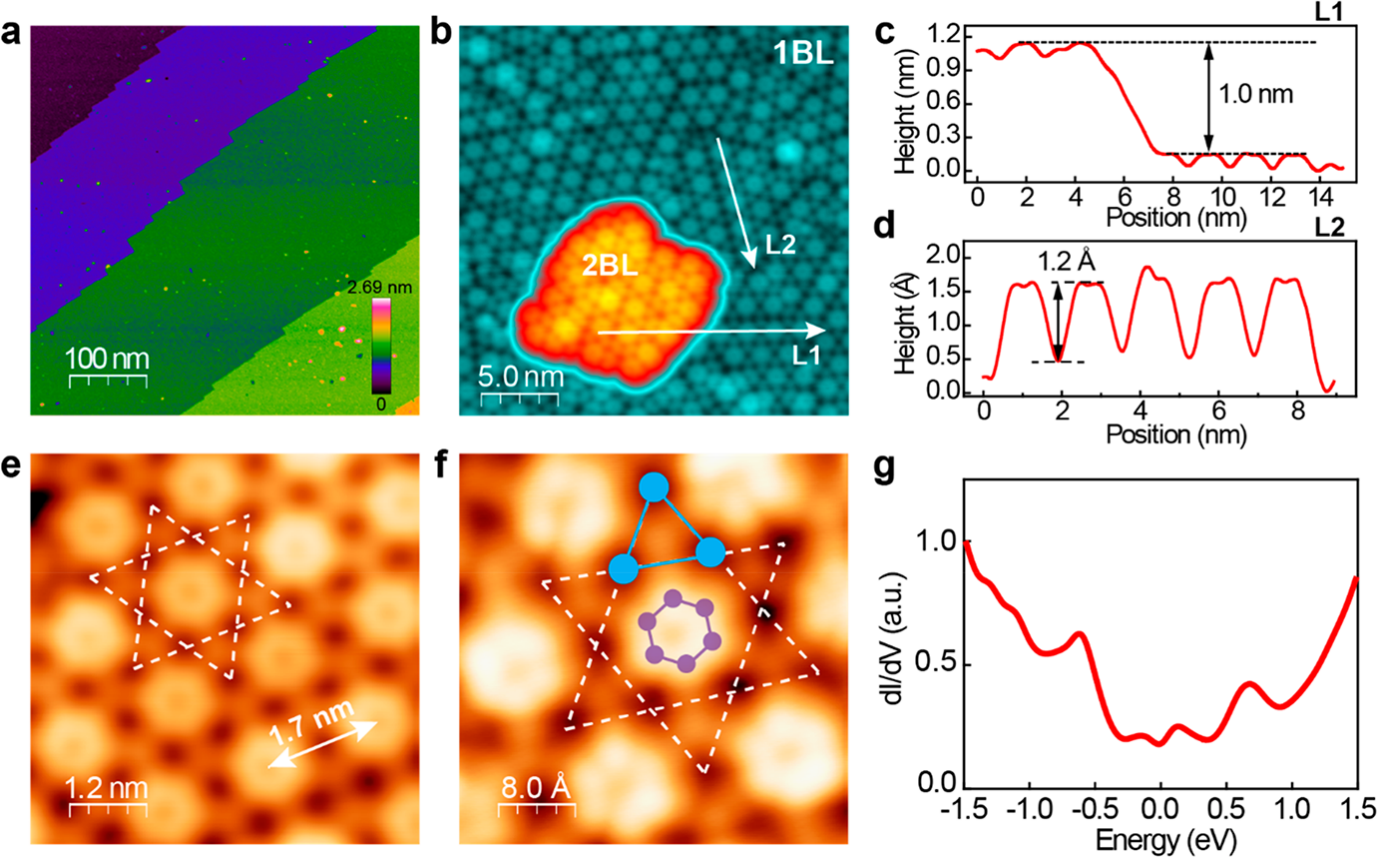
MBE-grown FeSb films on SrTiO_3_(001) substrate. (a) Topographic STM image of a 1 BL FeSb film epitaxially grown on the STO substrate. The step terraces in the image are from the STO substrate. Set point: *V* = 2.0 V, *I* = 10 pA. (b) Topographic STM image of a 2 BL FeSb island. Set point: *V* = 1.0 V, *I* = 50 pA. (c) Line profile along white arrow L1 in (b). The height of the 1 BL film is determined to be 1.0 nm. (d) Line profile along the white arrow labeled L2 in (b). The high-low contrast of the surface reconstruction is 1.2 Å. (e) Atomic resolution STM image of the 1 BL FeSb film showing a Kagome lattice. Set point: *V* = −0.1 V, *I* = 600 pA. (f) Close-up view of the Kagome lattice revealing the enhanced density of state at the center (purple hexagon) and the suppressed density of state at the shared triangle site (cyan triangle). Set point: *V* = 0.1 mV, *I* = 200 pA. (g) Typical d*I*/d*V* spectrum of the 1 BL FeSb film taken at the corner of the shared triangle site (cyan dot site in (f)).

Figure 1a presents a typical topographic STM image of FeSb films grown on Nb:SrTiO_3_ (STO) (001) substrates (see the Methods for growth conditions). The surface morphology of the FeSb film is conformal to the step-terrace topography of the STO substrate. The growth of FeSb film follows a layer-by-layer growth mode (Figure S1). The thickness of the FeSb film is determined to be ∼1.0 nm based on the analysis of line profiles as shown in Figure 1c, consistent with a bilayer FeSb (Supplementary Note 1, space group *P*6_3_/*mmc* with the lattice constant *a* = 0.4065 nm and *c* = 0.5121 nm). This is further confirmed by STEM images (Supplementary Note 2 and Figure S2), where the interface between FeSb and the TiO_2_-terminated STO is resolved with an epitaxial relationship of [11–20]FeSb//[100]STO. In particular, the two nearby Sb atom rows are clearly resolved with an Sb–Sb spacing of 4.1 Å, consistent with the theoretical value of 4.06 Å for FeSb. No discernible defects or extra Fe atoms in the film are observed in the STEM images.

The second bilayer grows only after the first bilayer film fully covers the STO substrate (Figure S1). We noted that the FeSb films exhibit different thermal stability, as the second bilayer starts to desorb when the substrate temperature increases to 438 °C (Figure S3). Moreover, thicker films beyond the second bilayer often exhibit a different structural phase (Figure S4). These observations suggest that the FeSb film is an interface-stabilized phase with a strong coupling between the first bilayer and the STO substrate, which is supported by DFT calculations, as discussed below.

The surface topography of the FeSb film exhibits multiple domains tens of nanometers in size (see STM images in Figure 1b and Figures S5 and S6), likely due to the growth of FeSb on STO(001) substrate that is symmetry mismatched with a 3-fold symmetric material epitaxially grown on the 4-fold symmetric STO (001) substrate (Figures S7 and S8). Consequently, a polydomain film is formed by multigrain coalescence during the growth.^34,35^

Within each domain, atomic resolution STM imaging reveals a Kagome lattice composed of hexagons and shared triangles denoted by the dashed pattern (Figure 1e,f, and Figure S5). The periodicity of the Kagome lattice is 1.7 nm, four times the lattice constant *a*_FeSb_ = 4.01 Å. Within the Kagome lattice, higher contrast is observed at the center, and lower contrast at the sharing triangle. From the line profile shown in Figure 1d, the high/low contrast gives rise to a difference of ∼1.2 Å, which exhibits minimum change under various bias *V* ranging from 1.0 V to −0.2 V (Figure S9). The Kagome structure observed here is similar to the previously reported superstructure formed by Fe deposited on the Sb(111) surface.^36^

## Electronic Properties of the Kagome Lattice

**Figure 2:**
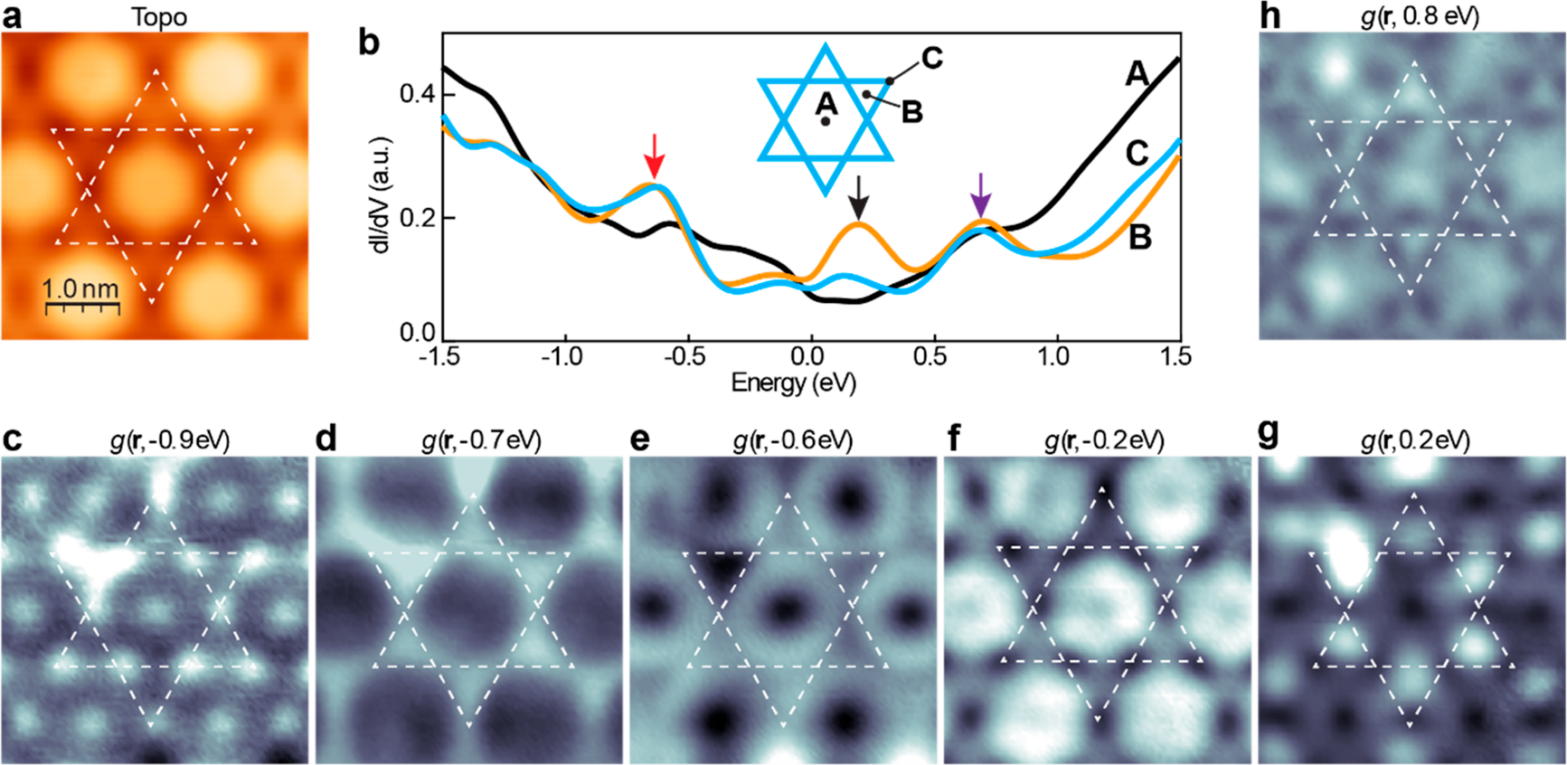
d*I*/d*V* maps of the Kagome lattice on the surface of the 1 BL FeSb/STO films. (a) Topographic STM image of the Kagome lattice. Set point: *V* = 1.0 V, *I* = 1.0 nA. (b) dI/dV spectra taken at the A (center), B (center of the shared triangle), and C (corner of the shared triangle) sites. The red, black, and purple arrows mark the peaks at −0.66, 0.18, and 0.69 eV, respectively. (c–h) Differential conductance maps at the energy specified. Set point: *V* = 1.0 V, *I* = 1.0 nA, and *V*_mod_ = 20 meV.

Multiple peaks are observed in the d*I*/d*V* spectrum in Figure 1g, and the nonzero density of states (DOS) at the Fermi level (*E*_F_) indicates a metallic behavior,^37,38^ which is also site-dependent (Figure S10). For the STM image in Figure 2a, d*I*/d*V* spectra were measured at three typical sites A, B, and C: the center of the hexagon, the center, and the corner of the shared triangle site, respectively (Figure 2b). Three pronounced peaks are marked by red (−0.66 eV), black (0.18 eV), and purple (0.69 eV) arrows in Figure 2b, indicating higher DOS from the shared triangle site (B or C) at these energies. This is directly reflected in the d*I*/d*V* maps shown in Figure 2c–h (and more data in Figure S11). The DOS is higher at the shared triangle corners, and the hexagon’s center for the map *g*(**r**, −0.9 eV) (Figure 2c). In Figure 2d, map *g*(**r**, −0.7 eV) highlights the shared triangle site. It was previously reported that higher DOS is observed at the shared triangle sites near the energy of the flat band in d*I*/d*V* maps of a Kagome lattice from a twisted silicene multilayer.^39^ Similarly, the peak at approximately −0.7 eV marked by the red arrow in Figure 2b likely corresponds to the flat band of the Kagome lattice on the surface of BL FeSb/STO. This is consistent with the map *g*(**r**, −0.7 eV), where the center of the Kagome lattice exhibits low contrast, whereas the shared triangle sites show higher contrast. For energies closer to the Fermi level, the maps *g*(**r**, −0.6 eV) (Figure 2e) and *g*(**r**, −0.2 eV) (Figure 2f) show structural details of the Kagome lattice’s center site, either a ring structure or a six-lobe feature. In contrast, above the Fermi level, the map *g*(**r**, 0.2 eV) (Figure 2g) has a higher DOS at the center of the shared triangle site. In the map *g*(**r**, 0.8 eV) (Figure 2h), the center site of the Kagome lattice exhibits a higher contrast. The appearance of the Kagome lattice is reproduced by DFT calculations, which will be discussed below.

## Magnetization Properties of 1 BL FeSb/STO Films

**Figure 3:**
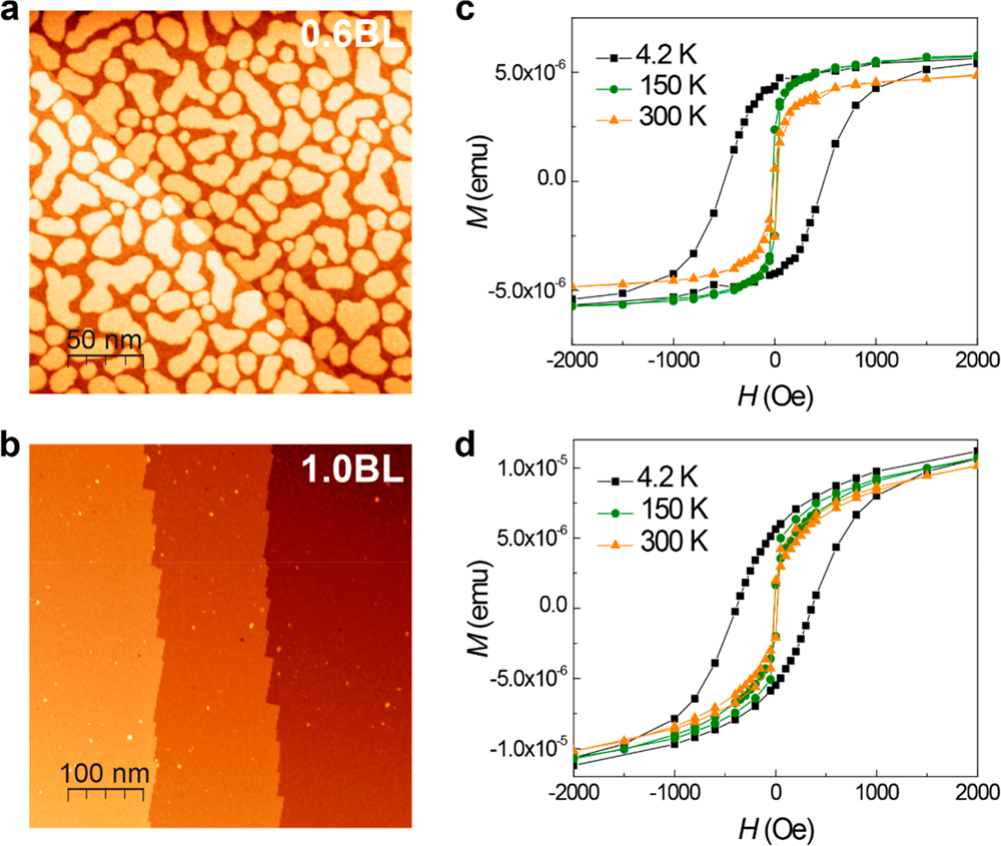
Magnetic properties of FeSb/STO(001) films. (a, b) Topographic STM images of FeSb films with coverages of 60% and 100%, set point: *V* = 3.0 V, *I* = 20 pA. (c, d) In-plane *M*–*H* taken at 4.2, 150, and 300 K for the two films in (a, b) (the magnetization saturates at *H* = 5000 Oe, see Figure S13 for details).

We performed *ex situ* magnetization measurements for FeSb/STO films after capping with ∼20 nm amorphous Sb. The results for two samples with 60% and 100% coverages (Figures 3a, b) are displayed in Figure 3c, d. Clear hysteresis is observed in the *M*–*H* plots. As the temperature increases from 4.2, 150 to 300 K, the coercive field decreases characteristic of ferromagnetic order. A hysteresis loop at 300 K is apparent, indicating a room-temperature ferromagnetism, consistent with temperature-dependent *M* vs *T* measurements (see Figure S12 for the *M*–*T* plots). A diamagnetic background is present in the raw data of the FeSb/STO films and was subtracted by corresponding linear fittings (Figure S13). Moreover, the FM signal is confirmed by the magnetization characterization of three additional 1 BL samples (Figure S14), where hysteresis are observable even at *T* = 390 K, indicating a Curie temperature *T*_C_ above 390 K. The external magnetic field, *H*, is applied parallel to the sample plane during the above measurements. In contrast, under out-of-plane magnetic fields, there are only small changes in the *M*–*H* loops (see Figure S15). Therefore, we conclude that the easy axis aligns along the in-plane direction. As the coverage increases from 60% to 100%, the coercive field *H*_c_ decreases from 485.3 to 374.7 Oe at *T* = 4.2 K, likely due to the film changing from isolated islands (single magnetic domains) at 60% coverage to percolated at full bilayer (Figure S6). The saturation magnetization *M*_*s*_ for 60% coverage is ∼5 × 10^–6^ emu (Figure 3c), comparable to the full bilayer film at ∼1.0 × 10^–5^ emu (Figure 3d), respectively.

We carried out control experiments to rule out extrinsic effects that could be responsible for the robust ferromagnetism presented above. First, the ∼20 nm Sb-capped STO substrate only shows a weak ferromagnetic order below 100 K and a diamagnetic order above 200 K (see Figure S16), consistent with that observed in SrTiO_3_ single crystal.^40^ Consequently, the ferromagnetic signal observed here originates from the epitaxial 1 BL FeSb/STO films rather than from the Sb capping layer or the STO substrate. In addition, the STO substrate only contributes to the diamagnetic background in the raw data of FeSb/STO films (Figure S13).

Next, we determined if excess Fe atoms in the film could contribute to the observed ferromagnetic order. This possibility can be ruled out based on the following two observations. First, no substantial extra Fe atoms or clusters are observed by STM (Figure S1) or STEM imaging (Figure S2). Second, we deposited Sb atoms onto the surface of the FeSb films and did not observe the formation of extra FeSb islands (Figure S17). If there were excess Fe atoms in the film, they might react with Sb and form additional FeSb islands, similar to the case of FeSe/STO^[41]^. This is also confirmed by the fact that the *M*_s_ is similar for FeSb films with coverages of 60% and 100%, because if the interstitial Fe atoms play a role, then the full bilayer film should exhibit a higher *M*_s_ than the film with 60% coverage. Based on the above, we conclude that ferromagnetism originates from the FeSb/STO films.

## Density-Functional Theory Calculations

**Figure 4:**
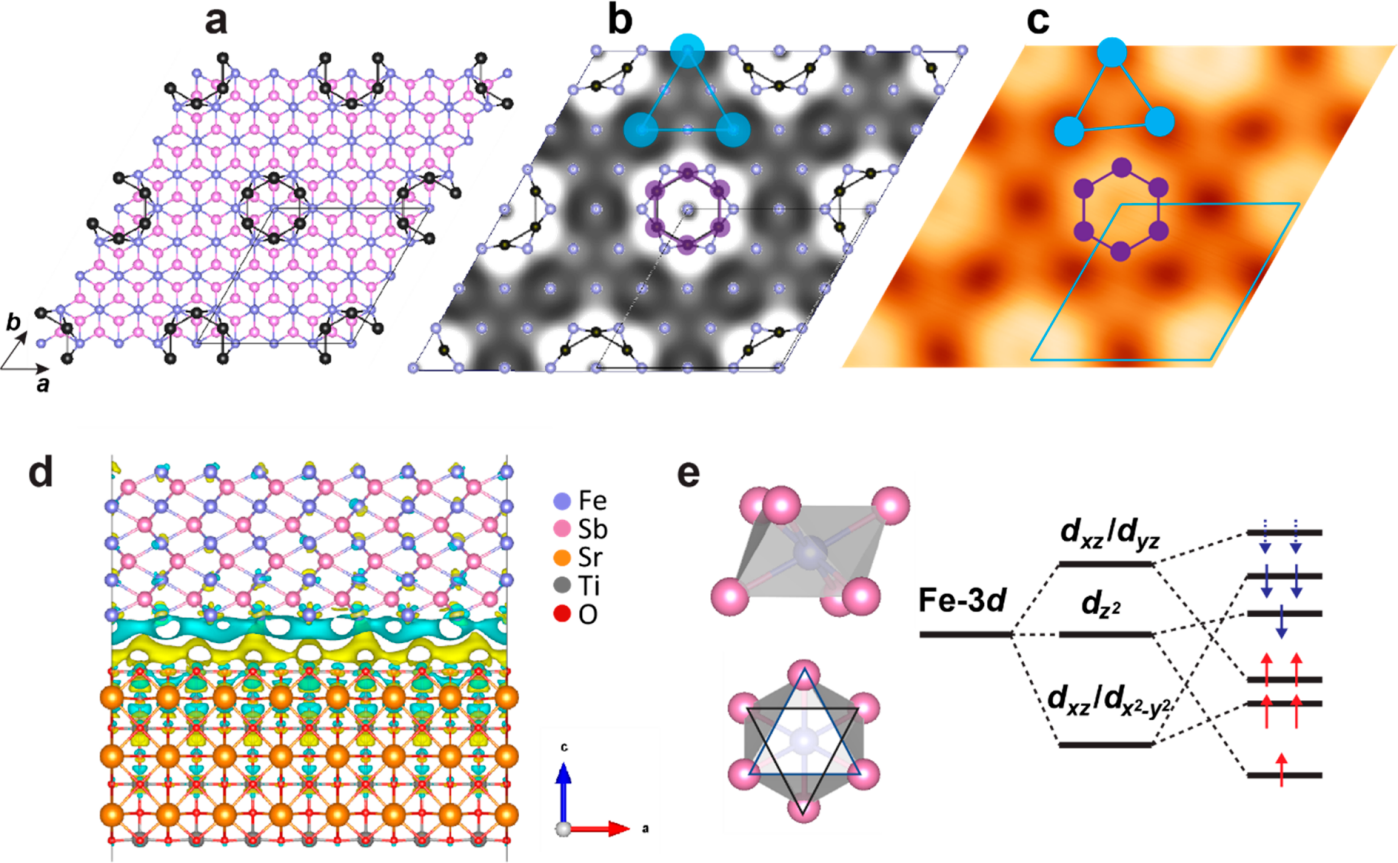
Density-functional theory calculations of the surface, interface, and magnetism in 1 BL FeSb/STO(001) films. (a) Ball-and-stick model of the Fe terminated bilayer FeSb film with Sb reconstruction (black atoms on top). (b) Simulated STM image of the configuration in (a) at *E* = 1.0 eV with the surface Fe and adsorbed Sb atoms marked. The shared triangle sites of the Kagome lattice are exactly at the Fe site, as denoted by the cyan triangle. (c) Experimental STM image showing enhanced DOS at the protrusion (hexagonal purple ring) and suppressed DOS at the hollow site (cyan triangle). Set point: *V* = −0.1 V, *I* = 600 pA. (d) Charge density of the FeSb/STO heterostructure. The isovalue is 0.0005 e Å^–3^. Fe, Sb, Sr, Ti, and O atoms are denoted by blue, pink, orange, gray, and red balls. The blue and yellow colors represent charge depletion and accumulation, respectively. (e) Schematic diagram of coordinated FeSb_6_ octahedron and the splitting of Fe-3d orbitals under a trigonal antiprismatic crystal field.

We further performed DFT calculations to determine the atomic arrangements of the FeSb films. Among all the six phases (Supplementary Note 1), our simulations indicate that only the ultrathin films with FeSb phase (space group *P*6_3_/*mmc* with the lattice constant *a* = 0.4065 nm and *c* = 0.5121 nm)^41,42^ are energetically favorable on TiO_2_-terminated SrTiO_3_(001) substrate. The thickness of the film ∼1.0 nm corresponds to bilayer FeSb (Figure S18). The epitaxial relationship between the FeSb and the TiO_2_-terminated STO substrate, their lattice mismatch, and the orientation of the FeSb polycrystals are shown in Supporting Information Note 3. We constructed the structure model based on DFT calculations and detailed analysis of the bias-dependent STM images. First, the topmost bright protrusions in the STM images consist of six bright features arranged in a regular hexagonal configuration, as shown in Figures S5 and S19c. Next, we calculated the adsorption energy (*E*_ab_) of Sb adatom on different sites of a bilayer FeSb, defined as , where *E*_total_ and  are the energies of the structure configuration with and without absorbed Sb atoms, and  is the energy of single Sb atom. Figure S19a and b show four typical sites labeled 1, 2, 3, and 4, and their corresponding adsorption energies (*E*_ab_) of the Sb adatom. Negative adsorption energy is observed for sites 1 and 2, with the lowest adsorption energy present at site 2 (−1.73 eV/unit), indicating that the energy will decrease with the Sb atom absorbed. We construct a surface adsorption structural model as shown in Figure 4a with site 2 adsorbed and find the corresponding simulated STM image (Figure 4b) perfectly fits the experimental one (Figure 4c). Both the enhanced DOS at the center (purple hexagon) and the suppressed DOS at the hollow site (cyan triangle) are reproduced. The calculated interface interaction energy *E*_inter_ of FeSb films exhibits a minimum for the bilayer (Supplementary Note 5 and Figure S18e), suggesting it is more energetically stable than the ML and TL, consistent with the bilayer growth observed by STM. Note that since FeSb is stacked alternatingly by Fe and Sb atomic layers along the *c*-direction, the film can either be Fe-terminated or Sb-terminated in principle (Supplementary Note 6). However, the reconstructed Sb-termination is triangular rather than hexagonal (Figure S20), which conflicts with the observation that STM imaging is independent of energy (Figure S9). Thus, the possibility of Sb termination is ruled out.

Overall, the adsorption of Sb atoms on the Fe-termination leads to the modulated electronic structure, which contributes to the Kagome lattice observed by STM, i.e., the Kagome layer is an Sb-induced reconstruction of the surface Fe atoms. This structure can host quantum states arising from the interplay of spin–orbit coupling, electron correlations, and topology.^43,44^ Our preliminary investigation has shown evidence of edge states (Figure S21), suggesting topological order in the BL FeSb/STO films.

## Insight into the Origin of Ferromagnetic Order in 1BL FeSb/STO Films

Based on DFT calculations, we discuss the intrinsic magnetism of FeSb films (Supporting Information, Note 7). In FeSb, each Fe atom is bonded to six equivalent Sb atoms to form FeSb_6_ octahedra, and the Fe-3*d* orbitals split due to a trigonal antiprismatic crystal field (Figure 4e). FeSb films exhibit long-range magnetic ordering, and the total magnetic moment *M* is contributed by Fe atoms. The calculated *M* is 2.04 μ_B_ per Fe atom for bilayer FeSb (Table S2), which is moderately smaller than the experimental estimation of 2.6 μ_B_ per Fe atom. The calculated magnetic anisotropy energy (MAE) is 0.412 meV per BL (Table S3). Interestingly, it favors in-plane anisotropy, consistent with the experimental observation. The bilayer FeSb is a metallic system evidenced by the calculated DOS (Figure S22). The calculated value of *D*(*E*_F_) × *I* is 3.17, which meets the Stoner criterion, i.e., *D*(*E*_F_) × *I* > 1 (Table S5), signifying itinerant ferromagnetism. Based on the mean-field Ising model, the calculated Curie temperature *T*_C_ of 1 BL FeSb and FeSb/STO film is 377.9 and 556.7 K (Supplementary Note 7), consistent with our experimental results (>390 K).

## Interfacial Effect in 1BL FeSb/STO Films

Figure 4d shows interfacial charge densities across the FeSb film (top) and the STO substrate (bottom) from the DFT calculations. Separation of blue (electron accumulation) and yellow (electron depletion) regions is revealed, indicating charge transfer between the STO and FeSb films. The charge transfer suggests a strong interaction between the FeSb films and the STO substrates. To address whether the accumulated charge is responsible for the emergent ferromagnetic order in FeSb/STO films, we grew FeSb films on double-layer graphene/SiC(0001) substrates, where a minimum interfacial effect is expected (Figure S23).^45,46^ Similar magnetic properties, including hysteresis loops persisting at T = 300 K, suggest that the ferromagnetic order is an intrinsic feature of the FeSb films rather than an interfacial effect. Moreover, we observed a slightly smaller *H*_c_ and *M*_s_ for FeSb films grown on the graphene/SiC(0001), which could be attributed to the more randomly distributed FeSb islands from the Volmer–Weber growth mode Figure S23) in contrast to the layer-by-layer growth on the STO substrate (Figure S1). The change of growth mode also confirms the much stronger interfacial interaction between the 1 BL FeSb/STO interface.

In summary, we successfully grew bilayer FeSb films on TiO_2_-terminated SrTiO_3_(001) substrates by MBE, an interface-stabilized 2D FM magnet terminated by an Fe Kagome layer. The films exhibit a ferromagnetic *T*_c_ > 390 K and an in-plane magnetic anisotropy, offering opportunities for further exploring emergent quantum phenomena from the interplay of ferromagnetism and topology with potential applications in spintronics.

## Methods

### Sample Preparation

The FeSb films were prepared by MBE on Nb-doped (0.05 wt %) SrTiO_3_(001) substrates. To achieve a flat surface with step-terrace morphology, the STO substrates were first degassed at 873 K for 3 h, followed by annealing at 1273 K for 1 h. High purity Fe (99.995%) and Sb (99.9999%) were evaporated from a homemade Tantalum boat and a K-cell onto the Nb-doped SrTiO_3_(001) substrate at temperatures between 573 and 623 K during growth with an estimated Sb/Fe ratio 5:1. The growth rate is 0.1 BL/min. The as-grown FeSb films were capped with ∼20 nm amorphous Sb films before being transferred out of the vacuum for magnetization measurements.

### Low-Temperature STM/S

Low-temperature STM/S measurements were carried out using a polycrystalline PtIr tip in a Unisoku-1300 STM system. The samples were transferred *in situ* to the STM chamber. During STM measurements, the sample was kept at *T* = 4.67 K with PtIr tips calibrated on Ag/Si(111) films. The STS was acquired using a standard lock-in technique with a bias modulation of *V*_rms_ = 20 mV at 732 Hz unless otherwise specified.

### Magnetization Measurement

The magnetic properties of the samples were measured in a Magnetic Property Measurement System (MPMSXL7) by Quantum Design with temperatures up to 390 K and magnetic fields up to 1000 Oe, and a Physical Property Measurement System (PPMS) with a VSM option by Quantum Design with a sensitivity of ∼10^–8^ emu.

### Density-Functional Theory Calculations

First-principles calculations were carried out with VASP. Spin-polarized generalized gradient approximation with Perdew-Burke-Enzerhof parametrization was used as the exchange-correlation functional, combined with projector-augmented wave pseudopotentials as well as a plane wave basis set with energy cutoff at 500 eV.^47^ Correction of van der Waals interactions using the DFT-D3 scheme was included in all calculations.^48^ A vacuum space of 20 Å thickness was added to avoid interaction between the FeSb slabs. During the calculations, a Monkhorst–Pack *k*-point mesh of 0.02 Å^–1^ was chosen for sampling the 2D Brillouin zones.

## Supporting Information Available

FeSb phase determination; scanning transmission electron microscopy characterization of FeSb/STO films; crystal orientation between FeSb and STO substrate; reference sample: 20 nm Sb/SrTiO_3_(001); thickness-dependent properties of FeSb; Fe termination vs Sb termination; first-principles calculations of magnetic parameters; comparison of FeSb with traditional ferromagnetic metals; layer-by-layer growth of FeSb films on STO(001) substrate; cross-sectional transmission electron microscopy (STEM) characterization of 1BL FeSb/STO film capped with amorphous Sb; epitaxial relationship between FeSb and STO substrate; energy-dependent d*I*/d*V* maps of the Kagome lattice; magnetization characterization of three additional 1BL FeSb/STO films; simulated STM images of bilayer FeSb with Fe-termination and Sb termination; edge states observed at the step edges of 2BL FeSb/STO(001) films (PDF)
