# Supplementary material for: Room-Temperature Ferromagnetism in Epitaxial Bilayer FeSb/SrTiO3(001) Terminated with a Kagome Lattice
Source: Nano Lett. 2023 Nov 1;24(1):122–9. doi: 10.1021/acs.nanolett.3c03415 (PMC10786153; doi:10.1021/acs.nanolett.3c03415)
Supplement: Supplementary file 1 — nl3c03415_si_001.pdf [file nl3c03415_si_001.pdf]

Room-temperature ferromagnetism in epitaxial bilayer FeSb/$SrTiO_3$(001) terminated with a Kagome lattice

Huimin Zhang,[1,2]†* Qinxi Liu,[3]† Liangzi Deng,[4]† Yanjun Ma,[1] Samira Daneshmandi,[4] Cheng Cen,[1,5] Chenyu Zhang,[6] Paul M. Voyles,[6] Xue Jiang,[2,3]* Jijun Zhao,[2,3] Ching-Wu Chu,[4] Zheng Gai[7], and Lian Li[1]*

*Corresponding author. Email: huiminzhang@dlut.edu.cn, jiangx@dlut.edu.cn, lian.li@mail.wvu.edu

This PDF file includes:

Supplementary Text
Figures S1 to S23
Tables S1 to S5
References

Supplementary Text

Note 1. FeSb phase determination

For bulk Fe-Sb, there are six crystal structures, as listed in Table S1, among which Fe_3Sb, Fe_4Sb_3, and FeSb exhibit hexagonal symmetry, while $FeSb_2$/$FeSb_3$ shows tetragonal/cubic symmetry. Based on the hexagonal symmetry revealed by the topographic STM images, we can rule out the possibility of two phases, $FeSb_2$ and $FeSb_3$. Moreover, our DFT calculations demonstrate that the other two phases, Fe_3Sb and Fe_4Sb_3, are not energetically stable on TiO_2-terminated $SrTiO_3$(001) substrate. Therefore, we determine that the only possible phase is the FeSb.

Note 2. Scanning transmission electron microscopy characterization of FeSb/STO films

The interface and epitaxial relationship between the FeSb and STO are determined by analysis of the atomic-resolution high-angle annular dark-field STEM (HAADF-STEM) images, as shown in Fig. S2. The STEM sample is capped with an amorphous Sb (*a*-Sb) layer, resulting in a heterostructure of *a*-Sb/bilayer FeSb/STO. Four Sb rows are expected for a bilayer for the FeSb with space group $P6_3/mmc$. In the STEM images, the interface between FeSb and the TiO_2-terminated STO (yellow arrow in Figs. S2c&d) is resolved with an epitaxial relationship of $[11\text{-}20]_{FeSb}//[100]_{STO}$. No discernible defects or extra Fe atoms in the film are spotted in the STEM images. The two nearby Sb atom rows are clearly resolved with an Sb-Sb spacing of 4.1 Å, consistent with the theoretical value of 4.06 Å for FeSb. While the third Sb row is still visible, the interface of FeSb and *a*-Sb is blurred. Thus, the 4th Sb is not resolved.

Note that the brightness of the Sb atom in Figs. S2c&d is much weaker than Sr even though Sb has a larger atomic number. This is likely due to the polycrystal nature of the BL FeSb films with a size of a single domain of roughly 10-20 nm (see Figs. S5-6) so that for typical TEM samples with five to ten domains with different crystalline orientations in the approximately 100 nm along the electron transmission direction. As a result, fewer Sb atoms are in the field of view during STEM imaging.

Cross-sectional FeSb/$SrTiO_3$(001) thin film samples were prepared by focused ion beam (FIB) liftout method using a Zeiss Ga-FIB to avoid oxidation while exposed to air. FIB final thinning was performed under 5 kV, 100 pA Ga ion beam, with further thinning inside a Fishione 1040 nanomill under 900 eV to get a thin sample and remove FIB damage. Samples were baked at 100

degrees under a high vacuum (10^{-8} Torr) overnight before being transferred into the TEM column. Standard plasma clean was not used to avoid possible oxidation of the sample. The sample was stabilized inside the TEM column for 4 hours to minimize sample drifting before image series were acquired. Scanning transmission electron microscopy measurements were performed on a probe-corrected FEI Titan STEM operated under 200 kV. Z-contrast high-angle annular dark field STEM images were acquired with a 24.5 mrad probe semi-convergence angle and 18.9 pA probe current. A Fishione 3000 HAADF detector was used, with inner and outer collection angles being 53.9 and 269.5 mrad, respectively. HAADF image series were obtained by acquiring 200 frames of 256 × 256 pixel HAADF images with 5 μs/pixel dwell time. The image series was processed by non-rigid registration[1] to compensate for the drift between frames before being averaged into one image with a high signal-to-noise ratio.

Note 3. Crystal orientation between FeSb and STO substrate

We show the schematic of the top view of the lattice alignment for FeSb and TiO_2 termination of the STO substrate in Fig. S7. The black rectangles in Figs. S7a and 6b denote the bonds of STO and FeSb are aligned in the same direction. The in-plane lattice constant for FeSb and STO is a_{FeSb} = 4.015 Å, a_{STO} = 3.905 Å. Considering the crystal orientation $[100]_{STO}$//$[11\text{-}20]_{FeSb}$, the lattice mismatch η along $[100]_{STO}$ and $[010]_{STO}$ can be calculated by the following equation:

$$\eta_{[100]STO} = \frac{4\sqrt{3}a_{FeSb} - 7a_{STO}}{7a_{STO}} = 1.76\%$$

$$\eta_{[010]STO} = \frac{a_{FeSb} - a_{STO}}{a_{STO}} = 2.82\%$$

Figure S6 shows STM images of FeSb/STO(001) films with different coverage. Note that for 0.6 BL (Fig. S6b) and 1.0 BL (Fig. S6f) FeSb, the ordered kagome lattice regions are comparable (marked by a dashed black outline), suggesting that the magnetic moment likely originates from these ordered kagome regions.

STO is cubic, so in-plane (100) and (010) are equivalent. The [1100]FeSb//[100]STO and [11-20]FeSb//[010]STO are equivalent, meaning in-plane multiple domain or polycrystal formation. As a result, we observe typical intersection angles between the kagome lattice with the STO[110], 30°, 45,° and 90°.

Note 4. Reference sample: 20 nm Sb/SrTiO$_3$(001)

The pristine SrTiO$_3$ single crystal is an ideal diamagnetic material due to the absence of unpaired electrons. Room-temperature ferromagnetism is reported in Nb-doped SrTiO$_3$ single crystals, where the ferromagnetic order is induced by oxygen vacancies and possibly mediated by free electrons from Nb doping[2]. The STO(001) substrates used in our experiments are annealed at 950 °C for 1h in an ultrahigh vacuum before epitaxial growth, which probably introduces oxygen vacancies in the STO substrate. The coercive field and the saturation magnetic moment in the 20 nm Sb capped STO(001) (308 Oe, 5×10^{-7} emu in Fig. S16) is smaller than that observed in bulk STO crystal (1 T, ~ 8.5×10^{-6} emu), which might be due to different oxygen vacancy density(*42*).

Note 5. Thickness-dependent properties of FeSb

The thickness of the epitaxial film measured from the STM image is ~1.0 nm, suggesting a bilayer FeSb (space group P6_3/mmc with the lattice constant *a* = 0.4065 nm and *c* = 0.5121 nm). We carried out thickness-dependent DFT calculations.

The formation energy E_f of the FeSb films is defined by the following:

$$E_f = E_{Fe_xSb_y} - xE_{Fe} - yE_{Sb} \qquad \text{(Equation 1)}$$

where E_{Fe} and E_{Sb} are the energy of each Fe and Sb atom in its most stable phase.
The interface interaction energy is defined as

$$E_{inter} = (E_{FeSb/STO} - E_{FeSb} - E_{STO})/S \qquad \text{(Equation 2)}$$

where $E_{FeSb/STO}$, $E_{FeSb,}$ and E_{STO} are the total energy of FeSb/STO superlattice, FeSb film, and STO substrate, respectively, and *S* is the cross-sectional area of FeSb/STO superlattice.
The formation energy E_f is higher for Fe-termination than Sb-termination. Take the bilayer as an example, E_f is 0.45 eV/atom for the Fe termination and 0.17 eV/atom for the Sb termination. Therefore, the Fe-termination is usually of the Sb atom reconstructed to reduce the formation energy of the system.
In Fig. S18e, we show that the interface interaction energy E_{inter} behaves differently for Fe-terminated and Sb-terminated films. As the thickness increases from monolayer (ML) to bilayer (BL) and triple layer (TL), the interaction energy shows a minimum at BL (-0.04385 eV/Å^2). It becomes comparably larger for ML (-0.04406 eV/Å^2), and TL (-0.02915 eV/Å^2). These results

suggest the bilayer is more energetically stable than the monolayer and triple layer and explains the bilayer growth mode observed by STM.

Note 6. Fe termination vs. Sb termination

Previous DFT calculations show that the Fe atom has a strong tendency to penetrate the Sb to form a highly coordinated configuration; on the contrary, the Sb atom prefers to stay above the substrate with a 3-coordinated structure[3], which favors an Sb-termination. The simulated STM images for Fe or Sb terminated bilayer FeSb are obtained by integrating the density of states and the results are displayed in Fig. S20. By carefully comparing the simulated STM images with experimental ones, we find that the Fe-termination is the optimal case (Fig. S20), where not only the enhanced DOS at the protrusion (purple hexagon in Fig. 4c) but also the suppressed DOS at the hollow site (cyan triangle in Fig. 4c) are reproduced. The reconstructed Sb-termination is found to show a triangular feature rather than a hexagonal protrusion at the center of the kagome in Fig. S20, which is inconsistent with the STM image in Fig. 4c. Therefore, the possibility of Sb-termination is ruled out.

Note 7. First-principles calculations of magnetic parameters

Using first-principles calculations, we discuss the intrinsic magnetic behavior of FeSb films. Firstly, the magnetic anisotropy energy (MAE) is defined as MAE = $E_{tot}[001] - E_{tot}[100]$, where $E_{tot}[001]$ and $E_{tot}[100]$ are to the total energies of states whose magnetization direction is perpendicular and parallel to the basal plane, respectively (Tables S2 and S3). The MAE is determined by considering the spin–orbit coupling through noncollinear calculations. The positive calculated MAE value indicates that the magnetization direction parallels the horizontal plane, consistent with the experimental observation.

Secondly, FeSb films exhibited long-range magnetic ordering, and the total magnetic moment is contributed mainly by Fe atoms. The magnetic moment calculated by DFT is 2.04 μ_B per Fe atom for the Fe-terminated bilayer (with stoichiometry Fe_5Sb_4 in Table S2). Simultaneously, we also evaluated the magnetic moment with corresponding experimental data by $M_s=M_{Fe}*N*(V_s/V)$, where M_s is the detected saturation magnetic moment 1.0×10^{-5} emu, N is the number of Fe atoms within the unit cell, and V_s or V represents the volume of the sample or unit cell, respectively. The estimated magnetic moment from experiments is 2.6 μ_B per Fe atom for Fe-terminated bilayer FeSb, which is slightly larger than that from DFT calculations.

As shown in Fig. 4e, each Fe atom sits inside the trigonal antiprismatic crystal field formed by neighboring six Sb atoms. The trigonal antiprismatic crystal field splits Fe 3*d* orbitals into d_{xz}/d_{yz}, d_{xy}/d_{x2-y2}, and d_{z2} states. Figure S22 further supports the 3*d*-orbital-resolved states of the Fe atoms in FeSb films in Fig. 4e. All *d* orbitals in the spin-up channel are fully occupied, and the d_{xy}/d_{x2-y2} and d_{z2} orbitals in spin-down state prefer to be occupied, while the d_{xz}/d_{yz} orbitals in spin-down state favor half-occupied. The unpaired d_{xz}/d_{yz} orbitals result in a magnetic moment of 1-2 μ_B in FeSb films. The calculated electron occupation number of the d_{xy}, d_{x2-y2}, d_{xz}, d_{yz}, and d_{z2} orbitals of Fe atoms in FeSb films are listed in Supplementary Table S4, further confirming Fe atoms' magnetic moments. The electron occupation number of occupied d_{xy}/d_{x2-y2} and d_{z2} orbitals in spin-down channel is 0.5-0.6, while the electron occupation number of half-occupied d_{xz}/d_{yz} orbitals in spin-down states is 0.3-0.4. Moreover, the difference between electron occupation number in spin-up and spin-down is 1.8-2.0, consistent with the magnetic moment values from DFT calculations. Thirdly, the origin of spontaneous magnetization in a metallic ferromagnet is generally accepted to be the itinerant electrons, which can be described by the well-known Stoner model. Therefore, we examine the Stoner criterion (Table S5). Stoner criterion is defined as $D(E_F)\times I$. Here $D(E_F)$ is the non-spin-polarized density of states at Fermi level (E_F), which is inversely proportional to the kinetic energy of electrons. The parameter *I* can be obtained by dividing the exchange splitting of spin-up and spin-down bands by the average magnetic moment. Indeed, these two Stoner parameters reflect the competition between exchange energy and kinetic energy. For metallic FeSb films, the calculated Stoner criterion is 3.17 for bilayer films, satisfying the condition $D(E_F)\times I > 1$, thus favoring the itinerant ferromagnetic ordering.

To calculate the Curie temperature T_C of the FeSb films, we used the mean-field Ising model, following the approach in earlier work[4,5]:

$$T_C = \frac{2}{3k_B}\frac{\Delta}{N}$$

where *N* is the number of magnetic atoms in the unit cell, k_B is the Boltzmann constant, and Δ is the total energy difference between the antiferromagnetic and ferromagnetic states of FeSb. With the AFM-FM energy difference of 0.049 and 0.072 eV per Fe atom, the calculated T_C are 377.9 K and 556.7 K for 1 BL FeSb and FeSb/STO films, consistent with our experimental results (> 390 K).

Note 8. Comparison of FeSb with traditional ferromagnetic metals

Owing to the nature of itinerant electrons, the Stoner model is able to illustrate the origin of the spontaneous FM order in both metallic Fe and FeSb ultrathin films. In our paper, we found that the transition temperature of FeSb is higher than that of ferromagnetic metals Fe, which mainly origin from the interface coupling effect. It is previously reported in traditional ferromagnetic metal, such as Fe and Co films, that the substrate morphology strongly influences the film and subsequently determine the electronic and magnetic properties in thin films, electronic transport, Curie temperature, coercive field, and anisotropies.

Despite high Currie temperatures in bulk Fe, Co, the T_c in thin films is dramatically lower than the bulk T_c and decreases distinctly with the film thickness in Co[6], Fe[7], and Ni[8]. Consequently, the T_c is far below 300 K, in great contrast to the high Currie temperature in FeSb which is well above 390 K, mainly due to the following three reasons:

(1) The higher Curie temperature in FeSb than that in ultrathin Fe and Co films is probably due to better interface perfection. As revealed by the STEM images (Fig. S2), the interface of FeSb/STO(001) is well aligned. In contrast, it is challenging to obtain a perfect interface in Fe, Co films. Take ultrathin Fe films as an example, its magnetic behavior is sensitive to both substrate surface morphology and surface stoichiometry, e.g. Fe thin films on GaAs substrates[9,10], and the decreased ferromagnetic behavior in ultrathin Fe thin film was attributed to Fe clusters[11]. All these reasons may largely reduce the strength of FM exchange interactions in Fe ultrathin films.

(2) The charge transfer from STO (001) substrate to the FeSb films (Fig. 4d), indicates that the ferromagnetism in FeSb films is enhanced by the electron doping imposed by STO substrate. The Stoner criterion implies that the itinerant ferromagnetism is mainly determined by the total density of states at the Fermi level. The extreme electron doping induced by the STO substrate causes a substantial shift in the electronic band of FeSb films. The variation in the density of states at the Fermi level, leading to an appreciable enhancement in the ferromagnetism. In the previous experimental work[12], Zhang et al. found that electron doping can boost T_c of Fe_3GeTe_2 to room temperature from ~30 K.

(3) The interface interaction can largely change the magnetic anisotropy energy (MAE). For ultrathin materials, MAE is a critical magnetic parameter of ferromagnets that counteracts thermal fluctuation and preserves long-range FM ordering. We performed MAE calculations

of Fe and FeSb thin films by DFT calculations and the results are shown in Table S3. In our DFT calculation, we considered ultrathin bcc Fe films with a thickness of 7 atomic layers (0.8 nm). The calculated MAE of ultrathin bcc Fe films is 31 μeV/Fe along the out-of-plane direction, which is close to the experimental results[13]. In contrast, the MAE in FeSb films ranges from 290 to 469 μeV/Fe depending on different termination and thickness, much larger than in Fe films.

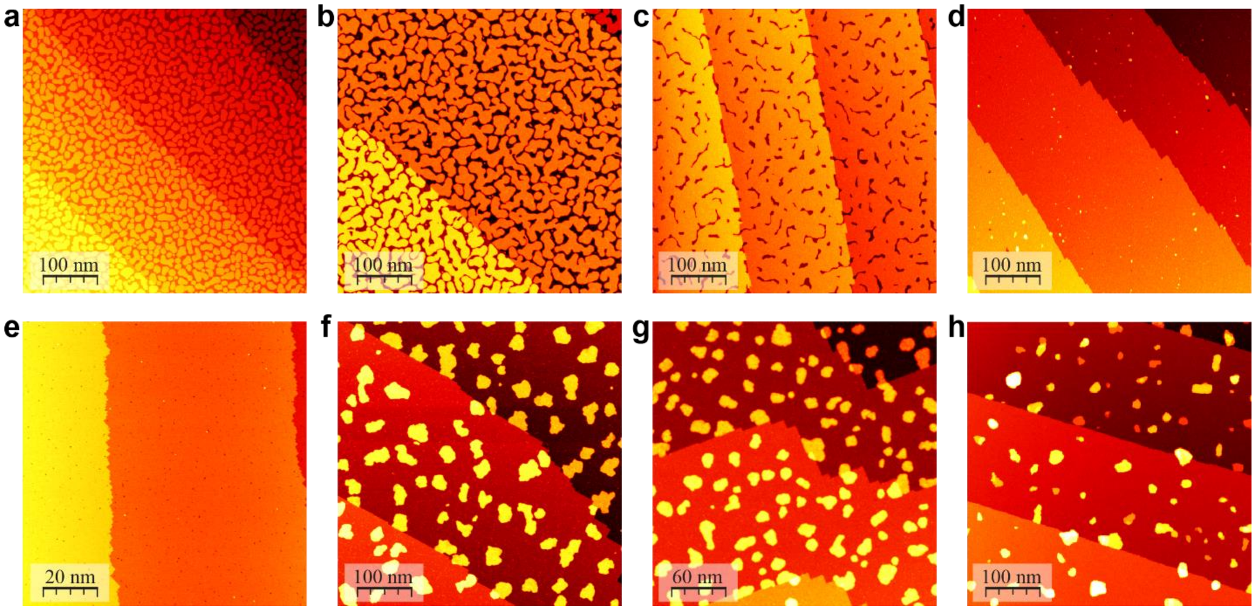

Figure S1. Layer-by-layer growth of FeSb films on STO(001) substrate. (a)-(g) Topographic STM images of FeSb/STO(001) films with different coverage θ. Setpoint: V = 3.0 V, I = 20 pA (a), V = 2.0 V, I = 10 pA (b), V = 3.0 V, I = 20 pA (c), V = 10.0 V, I = 10 pA (d), V = 4.0 V, I = 30 pA (e), V = 1.0 V, I = 1.0 nA (f), V = 3.0 V, I = 10 pA (g). (h) 1.2BL FeSb/STO(001) films after annealing at T = 438 °C for 20 min, where the 2nd BL film starts to decompose. Setpoint: V = 3.0 V, I = 20 pA.

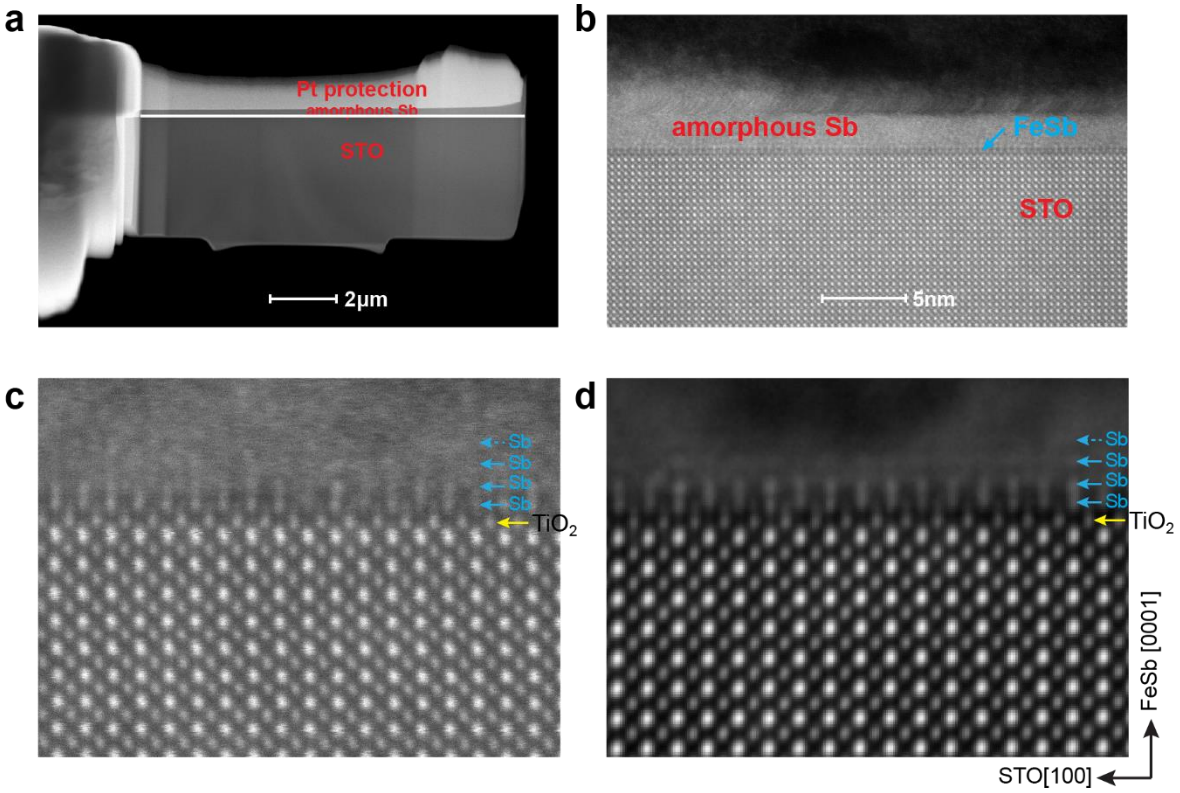

Figure S2. Cross-sectional transmission electron microscopy (STEM) characterization of 1BL FeSb/STO film capped with amorphous Sb. (a) FIB sample was lifted from the sample, ~11.6 μm in length. The sample is protected by Pt. (b) Zoom-in of the interface reveals homogenous FeSb film. (c) View under higher magnification. (d) Averaged from multiple registered images. The Sb atom rows are labeled by cyan arrows and the TiO_2 termination of the STO substrate is labeled by yellow arrow.

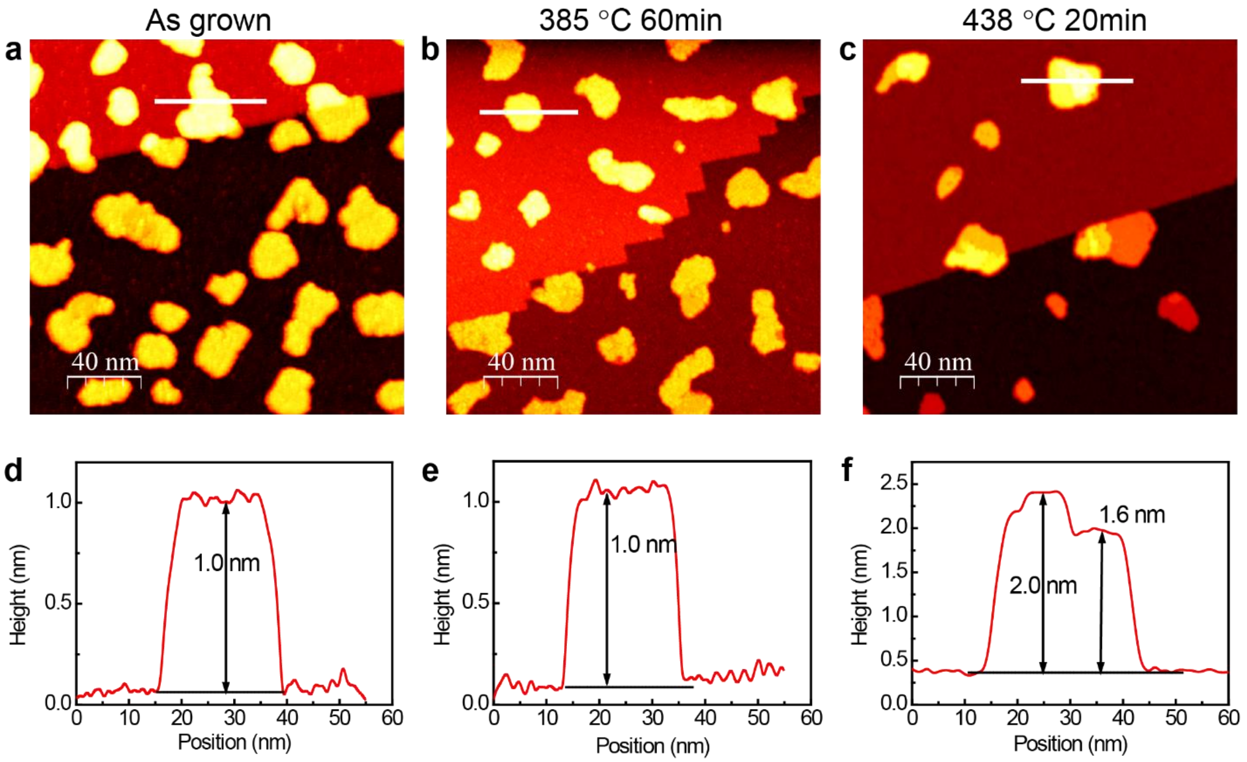

Figure S3. Instability of the 2nd BL FeSb films on STO(001) substrate. (a) Topographic STM images of as grown FeSb/STO(001) films at T_{sub} = 300 °C. Setpoint: V = 3.0 V, I = 20 pA. (b)-(c) Topographic STM images of the same sample after annealing at 385 °C for 60 min and 438 °C for 20 min, respectively. Setpoint: V = 3.0 V, I = 20 pA (b), and V = 2.0 V, I = 10 pA (c). (d)-(f) Line profiles along the white lines in (a)-(c). The 2nd BL island changes from flat-top (1.0 nm thick in (a) and (b)) to uneven surface (1.6-2.0 nm in (c)), suggesting the decomposition of the 2nd BL film at 438 °C. In contrast, the 1st BL film remains intact.

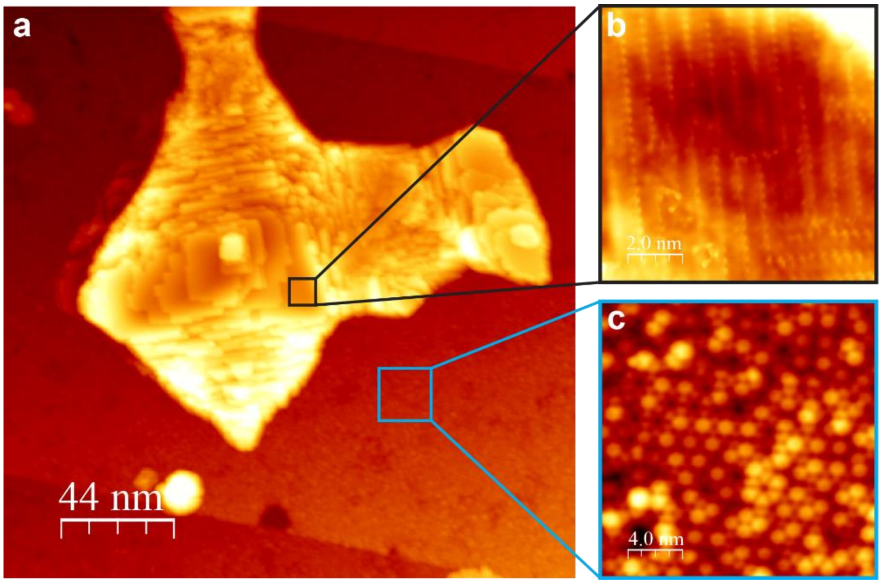

Figure S4. Different phases observed in thicker FeSb/STO(001) films. (a) Topographic STM images of thick FeSb/STO(001) films, where island growth is observed. Setpoint: V = 3.0 V, I = 50 pA. (b) Zoom-in region of the black box in (a). Setpoint: V = 50 mV, I = 1.0 nA. (C) Zoom-in region of the cyan box (bilayer) in (a). Setpoint: V = 1.0 V, I = 200 pA. Note that the bilayer FeSb remains intact, and thicker films exhibit a different phase.

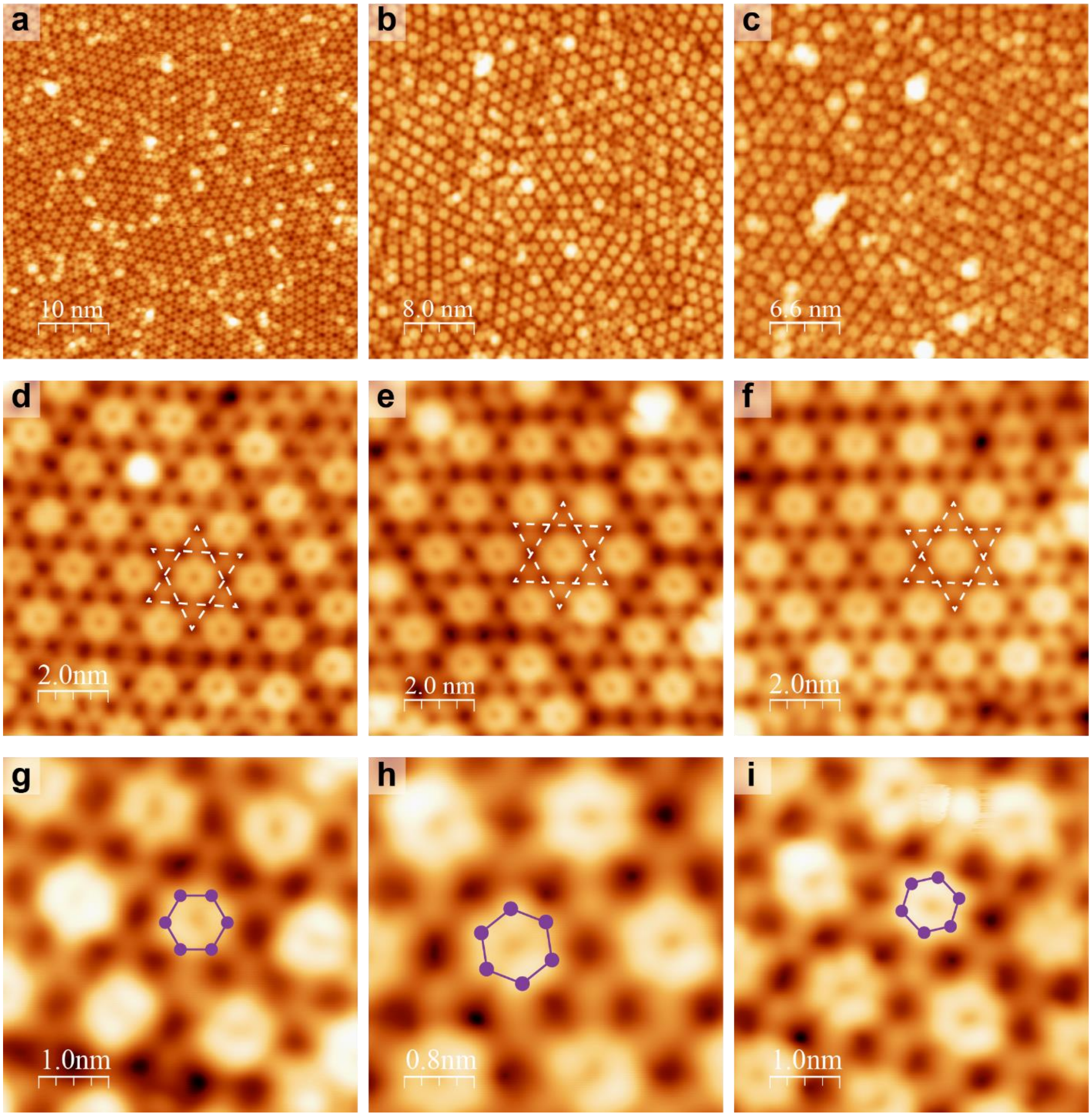

Figure S5. Kagome lattice in 1BL FeSb/STO(001) films. (a)-(c) Multidomain feature of the 1BL FeSb films. Setpoint: V = 1.0 V, I = 100 pA (a), V = -1.0 V, I = 0.5 nA (b), V = 1.0 V, I = 1.0 nA (c). (d)-(f) Kagome lattice inside single domain. Setpoint: V = 60 mV, I = 4.0 nA (d), V = 0.4 V, I = 1.0 nA (e), V = 0.3 V, I = 3.0 nA (f). (g)-(i) Protrusion at the center of the Kagome lattice with a honeycomb. Setpoint: V = 0.3 mV, I = 50 pA (g), V = 2.0 mV, I = 1.0 nA (h), V = 2.0 mV, I = 1.0 nA (i).

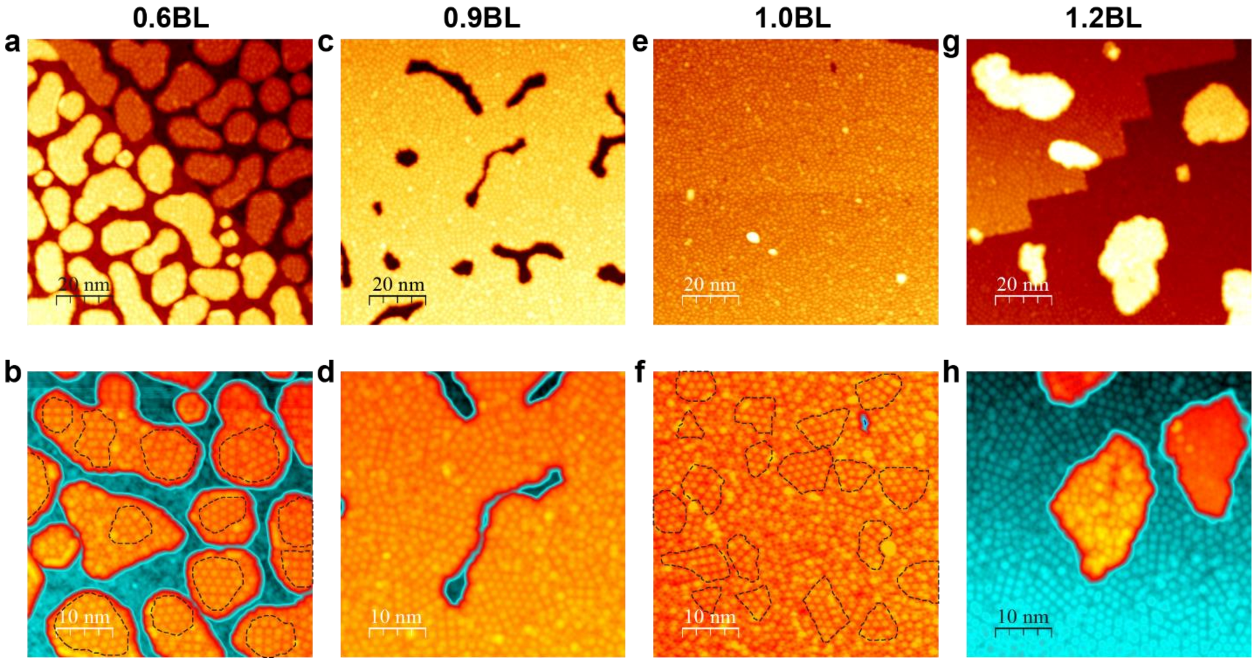

Figure S6. Ordered kagome lattice region in FeSb/STO(001) films with different coverage. (a)-(h) Topographic STM images of FeSb/STO(001) films with different coverage. Setpoint: V = 0.3 V, I = 20 pA (a), V = 0.2 V, I = 100 pA (b), V = 1.0 V, I = 10 pA (c), V = 1.0 V, I = 10 pA (d), V = 4.0 V, I = 30 pA (e), V = 1.0 V, I = 1.0 nA (f), V = 0.5 V, I = 100 pA (g), and V = 0.5 V, I = 100 pA (h). The dashed black outline in (b) and (f) marks the ordered kagome region in 0.6BL and 1.0BL.

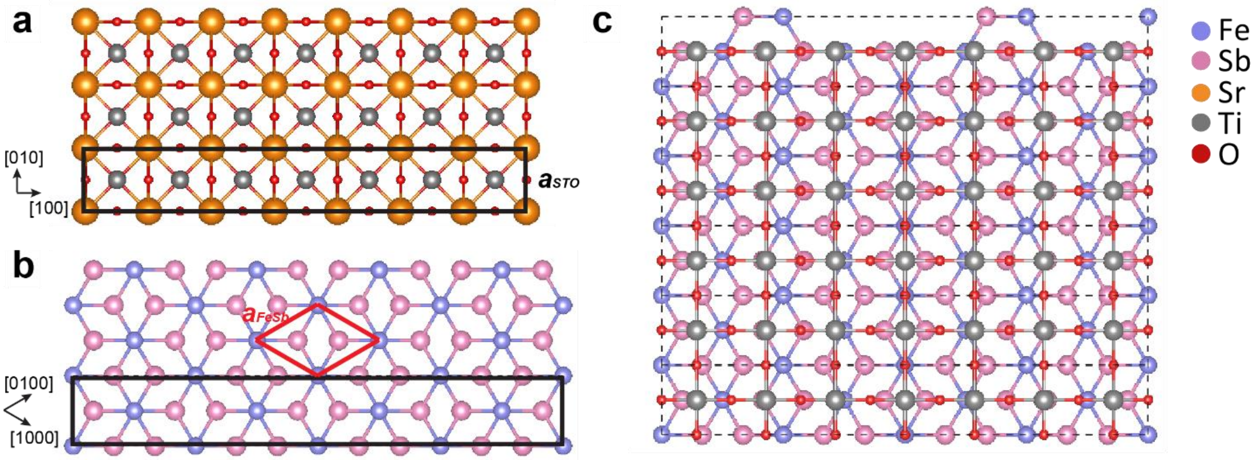

Figure S7. Schematic lattice alignment for FeSb and STO. (a) Crystal structure of STO from the top view. (b) Crystal structure of FeSb from the top view. (c) Lattice alignment between FeSb and the TiO_2 termination of the STO substrate from the top view.

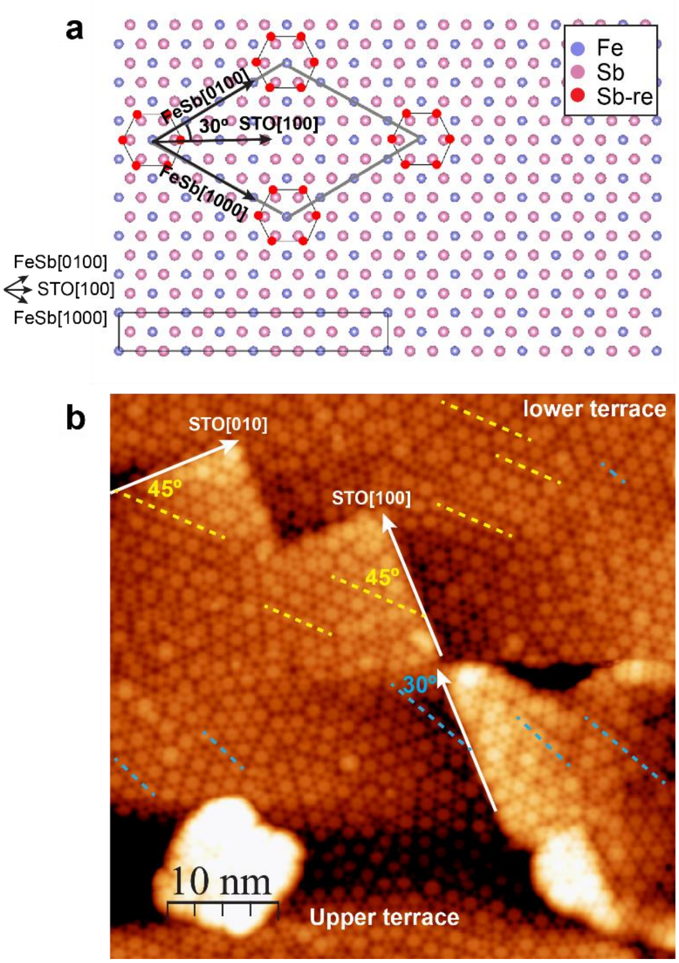

Figure S8. Epitaxial relationship between FeSb and STO substrate. (a) Schematic crystal structure of FeSb from the top view. (b) Topographic STM image of FeSb/STO(001) films. Setpoint: V = 0.1 V, I = 50 pA. The white arrow denotes the step terrace of STO. The yellow and cyan dashed lines denote two main orientations.

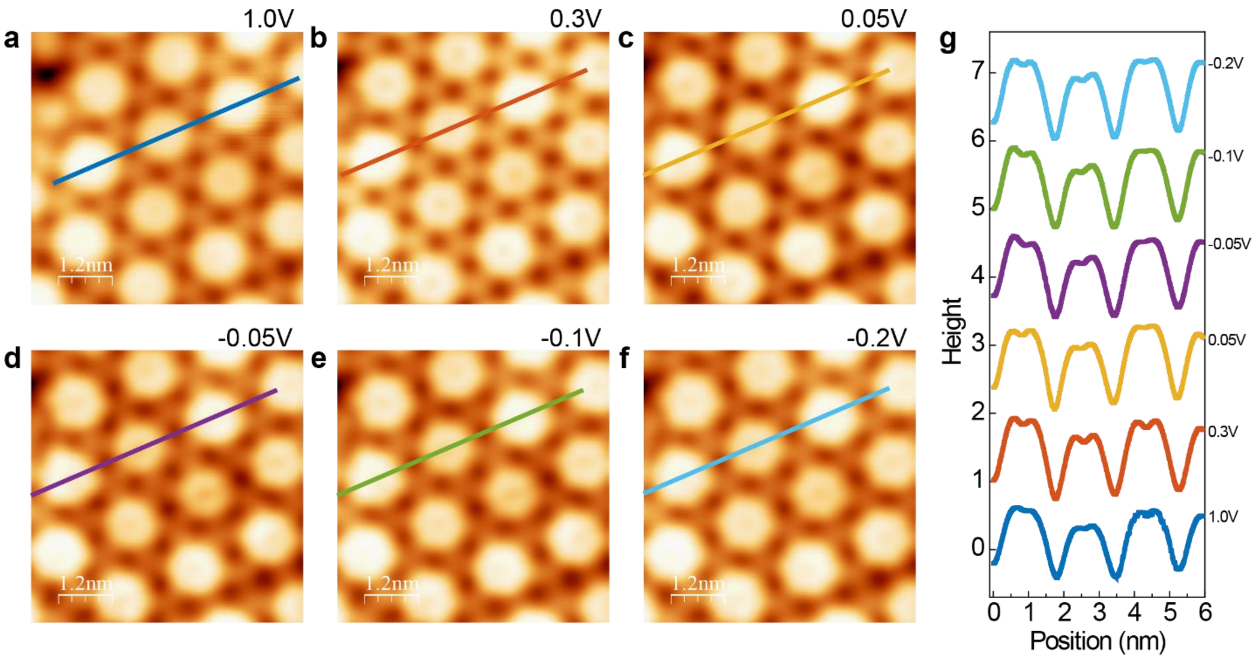

Figure S9. Bias-dependence of the Kagome lattice in 1BL FeSb/STO(001) films. (a)-(f) Topographic STM images of the Kagome lattice under various bias *V* specified. Setpoint: *I* = 5.0 nA. (g) Line profiles across the lines in (a)-(f), respectively. The curves are offset vertically for clarification. Little change is observed in the line profile under different bias *V*.

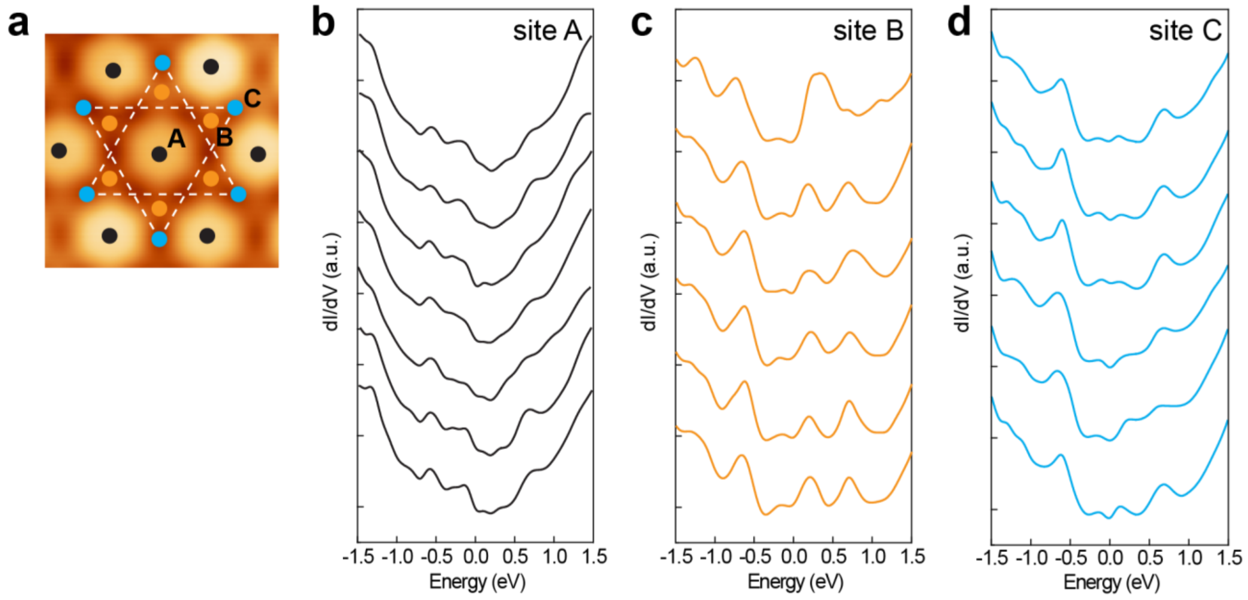

Figure S10. dI/dV spectra of 1BL FeSb/STO(001). (a) Topographic STM image of the Kagome lattice. Setpoint: V = 1.0 V, I = 1.0 nA. Size: 5×5 nm^2. The black, orange, and cyan dots mark the center (A), the center (B), and corner (C) of the shared triangle sites. (b) dI/dV spectra taken at the A, B, and C sites, respectively.

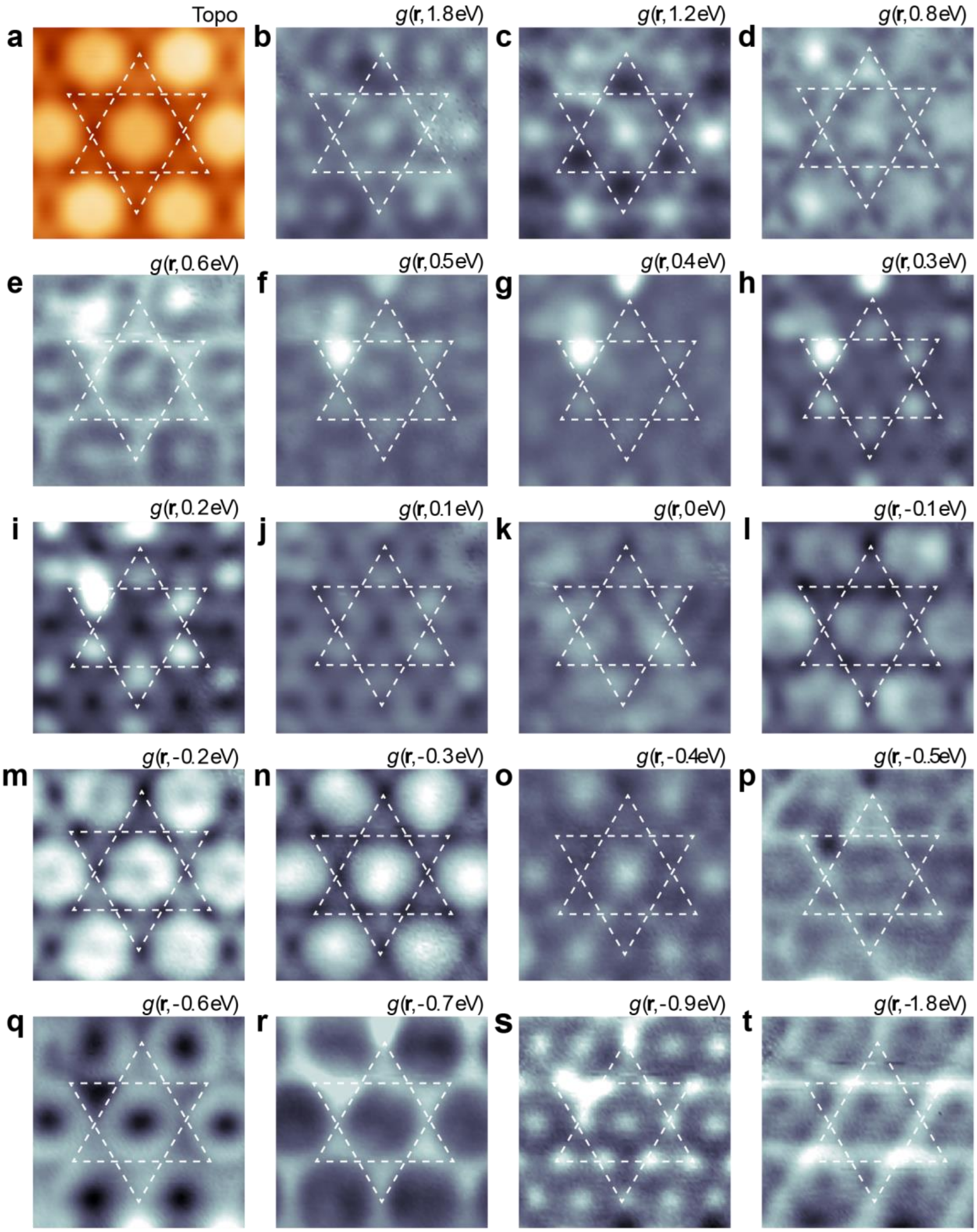

Figure S11. Energy-dependent dI/dV maps of the Kagome lattice. (a) Topographic STM image showing the Kagome lattice. Setpoint: V = 1.0 V, I = 1.0 nA. (b)-(t) dI/dV maps at the energy specified. Setpoint: V = 1.0 V, I = 1.0 nA, V_{mod} = 20 meV.

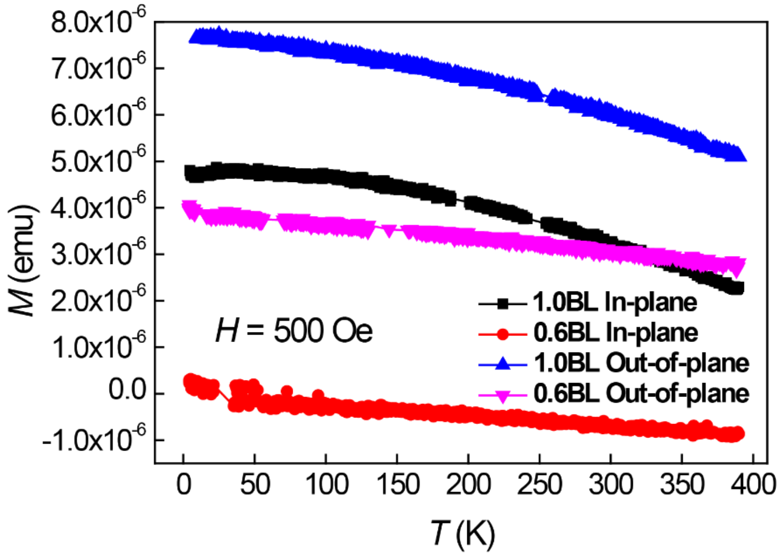

Figure S12. Temperature-dependent in-plane versus out-of-plane magnetic properties of FeSb/STO films. In-plane and out-of-plane *M*-*T* curves under a magnetic field of 500 Oe (before diamagnetic background deduction) for the two films shown in Figs. 3a&b.

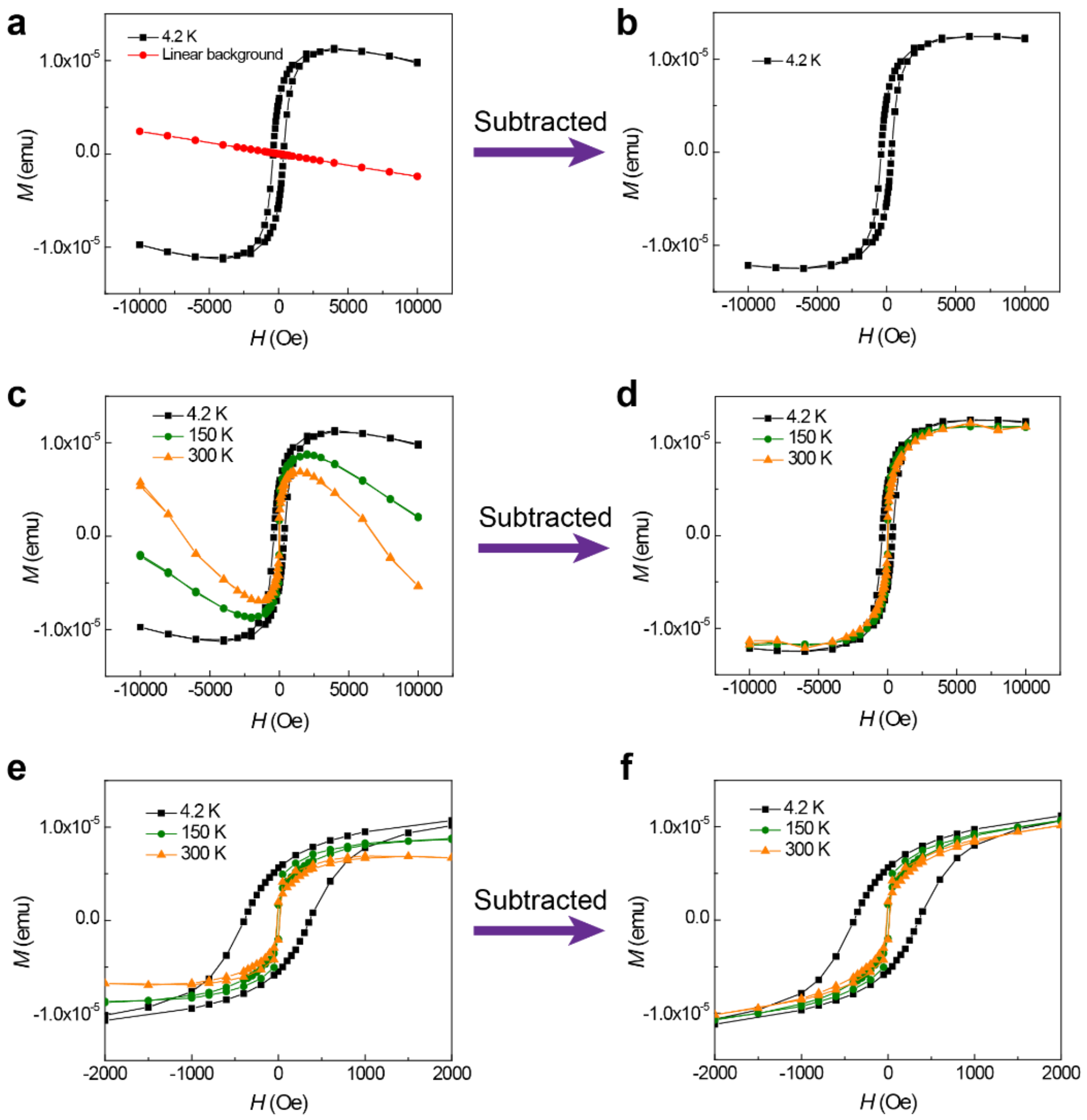

Figure S13. Background subtraction of 1BL FeSb/STO films. (a) *M*-*H* curves at T = 4.2 K and corresponding linear background. (b) *M*-*H* curves with background subtracted. (c)-(d) original *M*-*H* loops and corresponding ones after linear background subtracted. (e)-(f) Zoom-in of (c)-(d).

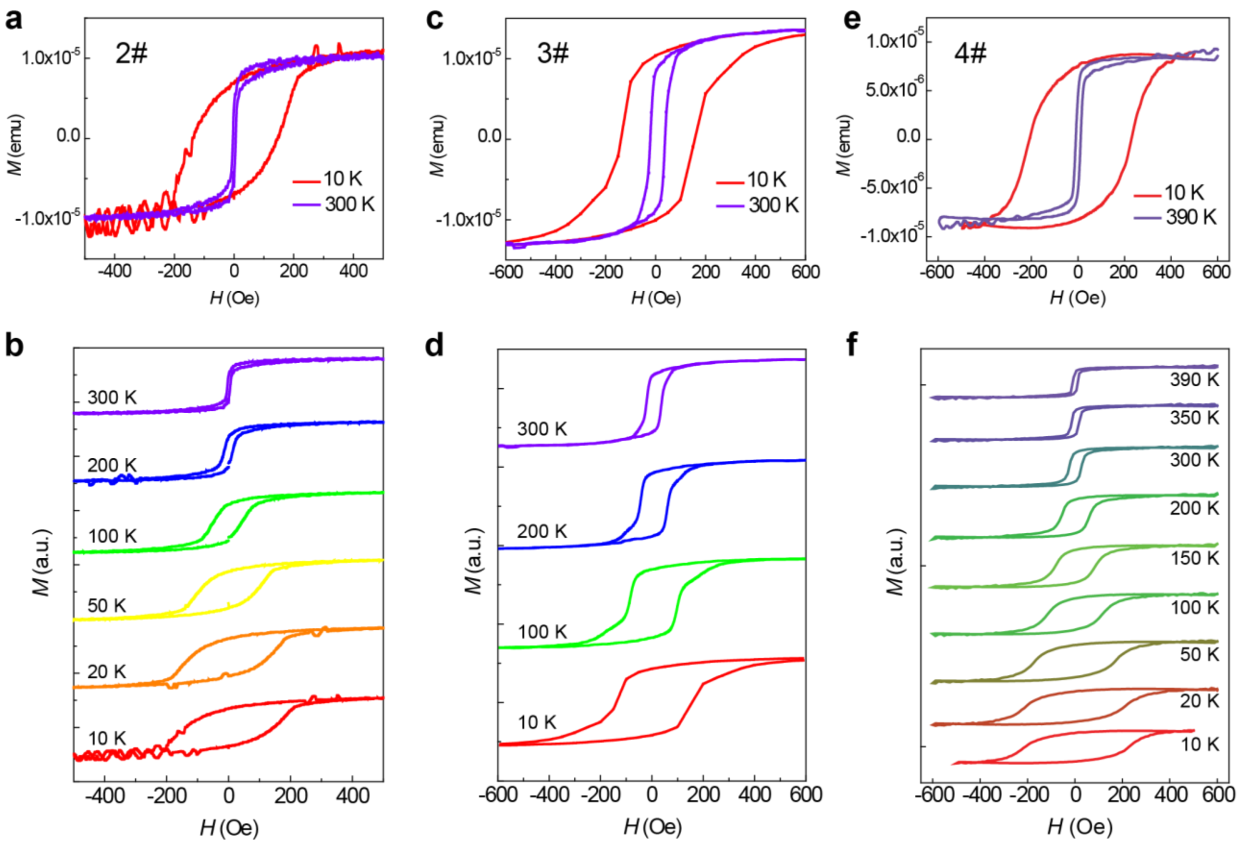

Figure S14. Magnetization characterization of three additional 1BL FeSb/STO films. (a)-(b) *M-H* curves for sample 2# measured from 10 K to 300 K. (c)-(d) *M-H* curves for sample 3# measured from 10 K to 300 K. (e)-(f) *M-H* curves for sample 4# measured from 10 K to 390 K. The capping layer is ~ 20 nm thick amorphous Sb and Bi_2Te_3 for sample 2# - 3#, and 4#, respectively.

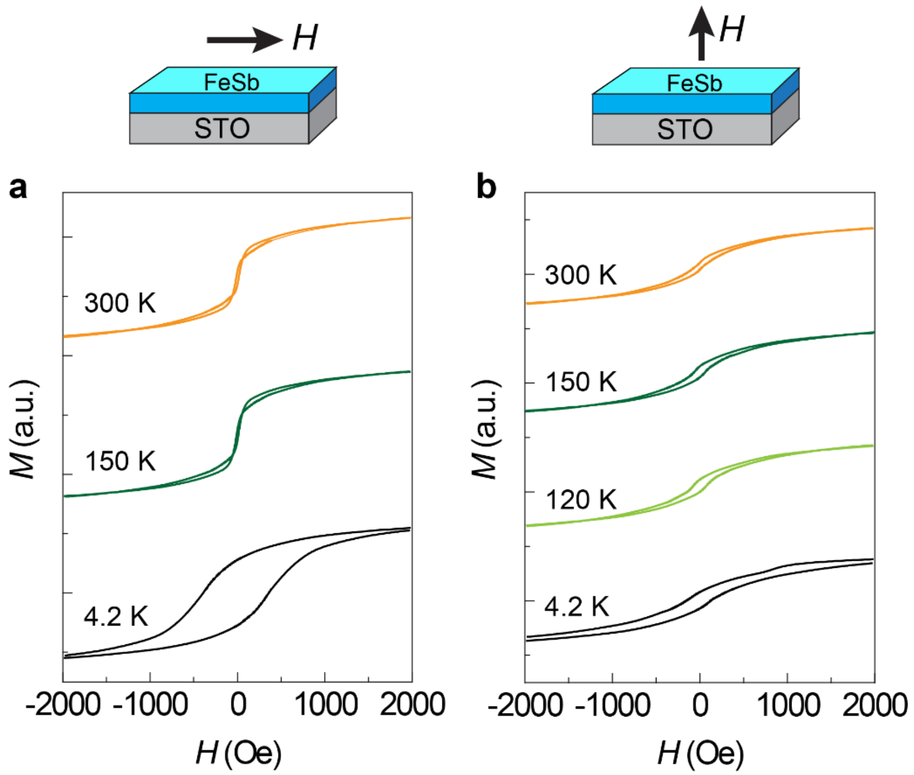

Figure S15. In-plane versus out-of-plane magnetic properties of 1.0 BL FeSb/STO film in Fig. 3d. (a) In-plane *M-H* hysteresis loops under 4.2 K, 150 K and 300 K as specified. (b) Out-of-plane *M-H* hysteresis loops under 4.2 K, 120 K, 150 K and 300 K as specified. We noticed that under out-of-plane *H*, the coercive field (H_c) changes minor with temperature (H_c = 137.7 Oe at T = 4.2 K), which is in great contrast with that under in-plane magnetic field where H_c shrinks with increased temperature, the maximum H_c = 370.5 Oe at T = 4.2 K. The saturation magnetization at out-of-plane direction is comparable to that under in-plane, $M_s \sim 1.0 \times 10^{-5}$ emu. The magnetic properties were measured in a Physical Property Measurement System (PPMS) with a VSM option by Quantum Design.

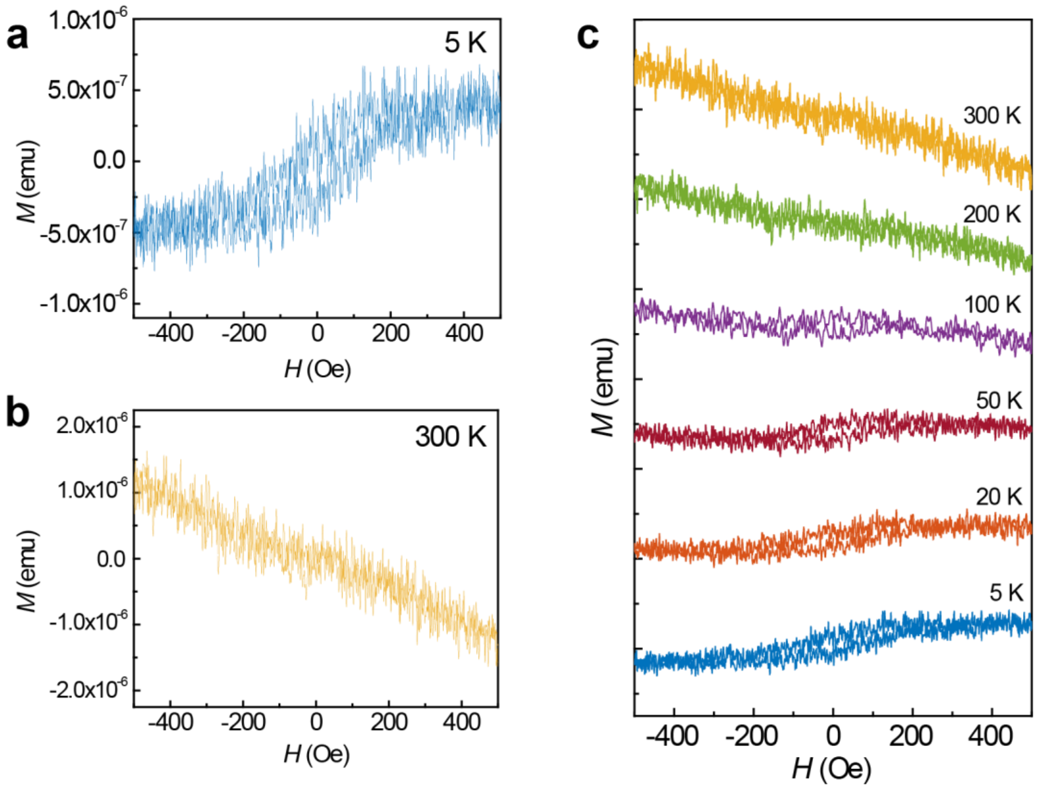

Figure S16. Magnetic characterization of the reference sample 20nm Sb/STO(001) film. (a) *M-H* curve measured at $T = 5$ K via VSM, showing a weak ferromagnetic order with cohesive field $H_c = 308$ Oe and saturation magnetization $M_z = 5\times10^{-7}$ emu. (b) *M-H* curve measured at $T = 300$ K, representing a diamagnetic order. (c) *M-H* hysteresis loops measured at various temperatures. Ferromagnetic order is observed at $T < 100$ K and diamagnetic order appears at $T \geq 200$ K. The transition temperature from ferromagnetic order to diamagnetic order is between 100 K and 200 K. The magnetic properties were measured in a Physical Property Measurement System (PPMS) with a VSM option by Quantum Design.

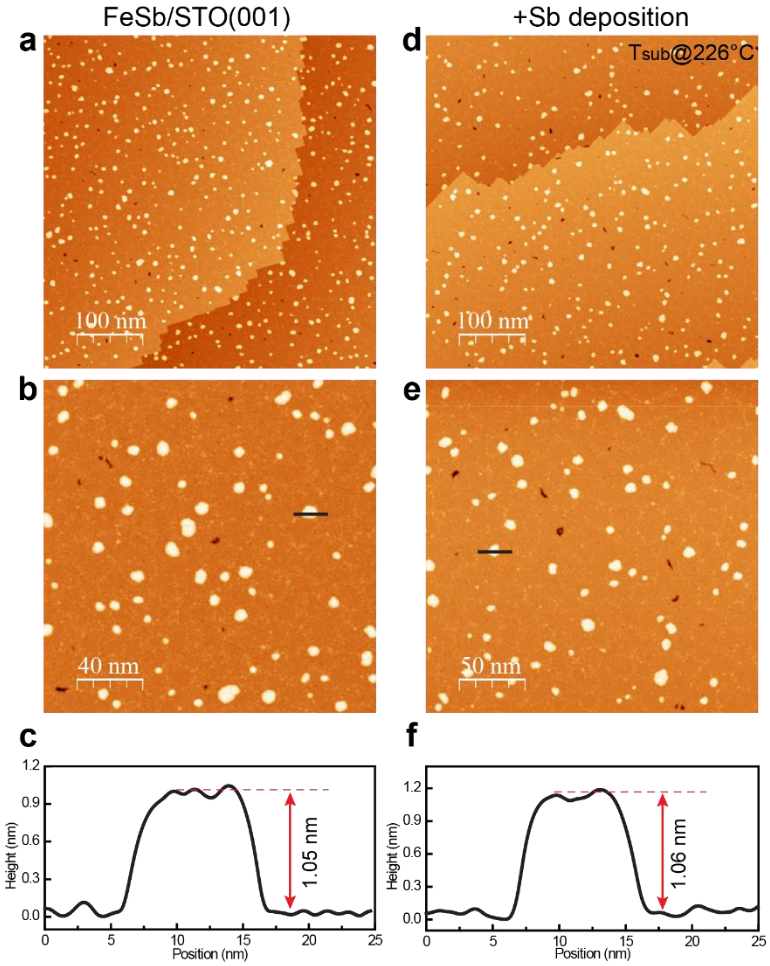

Figure S17. Reference experiments: to deposit Sb onto FeSb/STO films. (a)-(b) Topographic STM images of 1.04BL FeSb/STO(001) films. Setpoint: V = 2.0 V, I = 20 pA (a), and V = 2.0 V, I = 20 pA (b). (c) Line profile across the black line in (b) showing the thickness of the island h = 1.05 nm. (d)-(e) Fe deposited on Sb(111) films. Setpoint: V = 2.0 V, I = 10 pA (d) and V = 2.0 V, I = 10 pA (e). The coverage is determined to be 1.04 BL and does not change. (c) Line profile across the black line in (d).

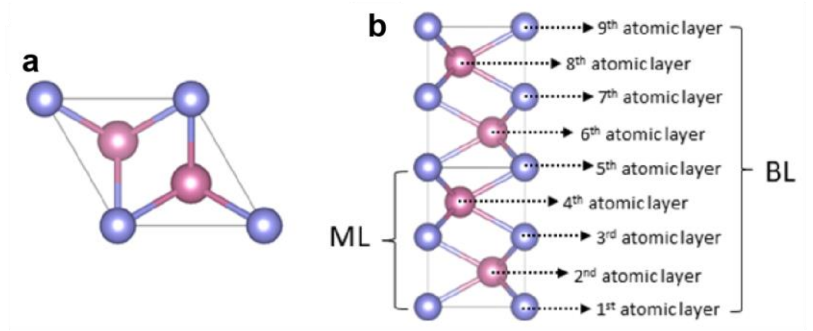

c

	monolayer (ML)		bilayer (BL)		triple-layer (TL)
	Fe-ter	Sb-ter	Fe-ter	Sb-ter	Fe-ter
Height (Å)	Fe_3Sb_2	Fe_2Sb_3	Fe_5Sb_4	Fe_4Sb_5	Fe_7Sb_7
	6.4	6.5	11.1	11.4	17.4

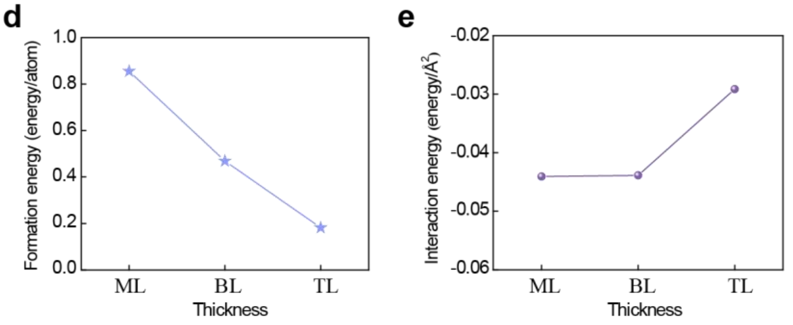

Figure S18. DFT calculations of thickness-dependent FeSb films. (a)-(b) Schematic crystal structure of FeSb from the top view and side view. (b) Height of FeSb films with different thicknesses. (c) The formation energy of monolayer (ML), bilayer (BL), and triple layer (TL) Fe-terminated FeSb films. The formation energy is defined as $E_f = E_{Fe_xSb_y} - xE_{Fe} - yE_{Sb}$, where E_{Fe} and E_{Sb} are the energy of each Fe and Sb atom in its most stable phase. (d) Interface interaction energy between the STO substrate and Fe-terminated FeSb films. The interface interaction energy is defined as $E_{inter} = (E_{FeSb/STO} - E_{FeSb} - E_{STO})/S$, where $E_{FeSb/STO}$, E_{FeSb} and E_{STO} are the total energy of FeSb/STO superlattice, FeSb film and STO substrate, respectively, and S is the cross-sectional area of FeSb/STO superlattice.

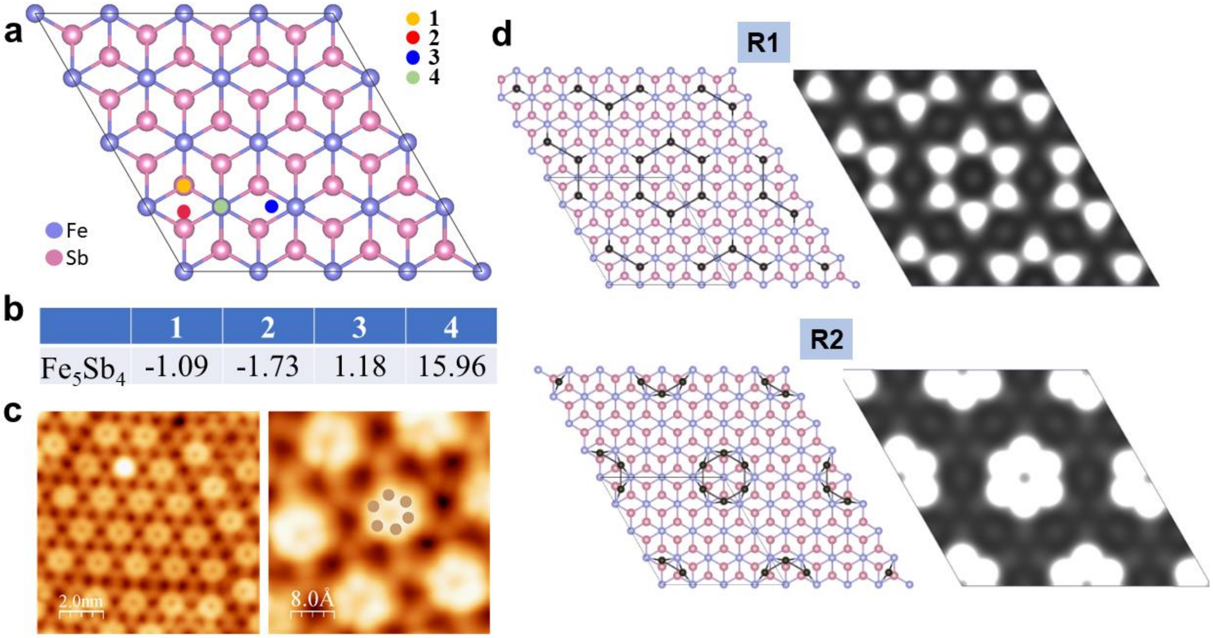

Figure S19. Structure configuration of FeSb films. (a) Four typical adsorption configurations of Sb atom on Fe_5Sb_4. (b) The calculated adsorption energy E_{ab}(eV/unit) of one Sb atom on FeSb films. The adsorption energy is defined as $E_{ab} = E_{total} - E_{film}^{FeSb} - E_{atom}^{Sb}$, where E_{total} and E_{film}^{FeSb} are the energy of the adsorption configurations with and without absorbed Sb atom. E_{atom}^{Sb} is the energy of a single Sb atom. (c) Topographic STM images show that the surface reconstruction consists of six Sb atoms. (d) Simulated STM images with two surface reconstruction configurations, R1 and R2 with surface Sb atoms located at site 1 and 2, respectively.

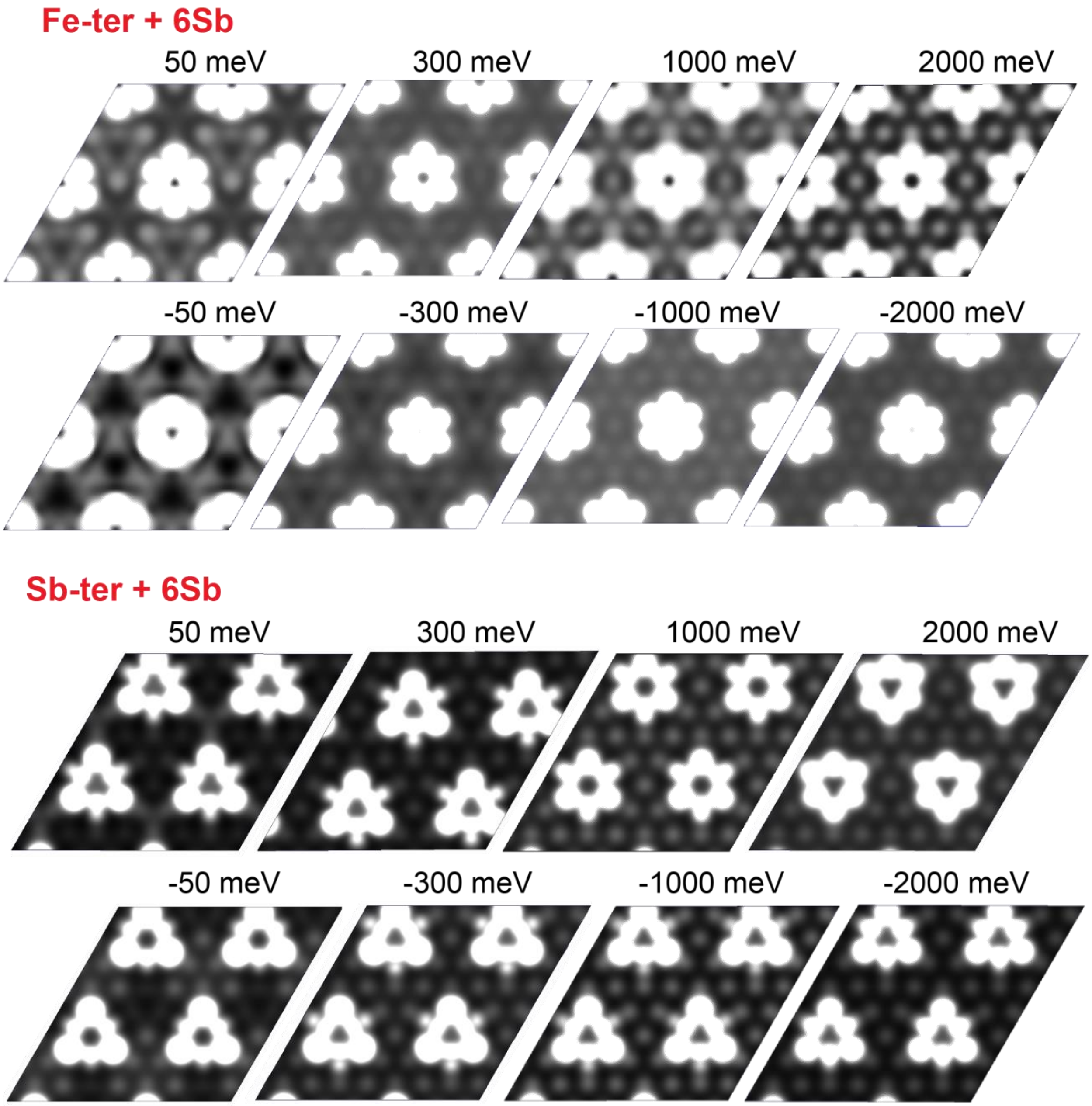

Figure S20. Simulated STM images of bilayer FeSb with Fe-termination and Sb termination. The energy is specified.

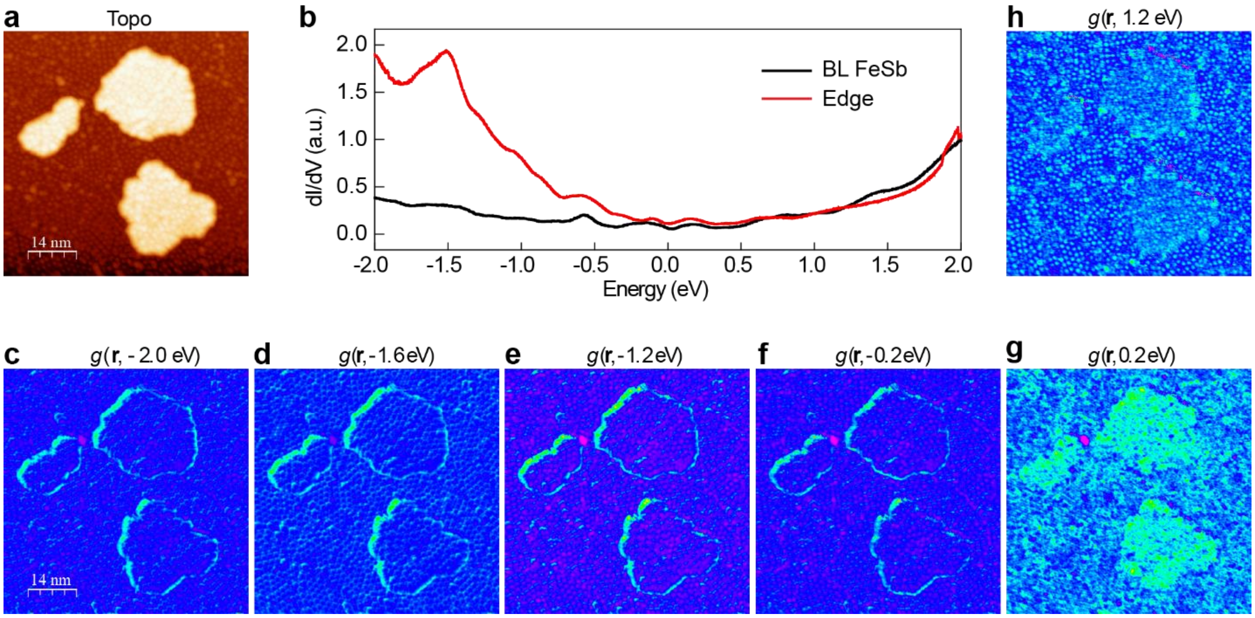

Figure S21. Edge states observed at the step edges of 2BL FeSb/STO(001) films. (a) Topographic STM image of three 2BL FeSb islands. Setpoint: V = 2.0 V, I = 0.3 nA. (b) dI/dV spectra taken at bilayer FeSb and the edges of 2nd BL FeSb films. (c)-(h) Differential conductance maps at the energy specified. Setpoint: V = 2.0 V, I = 0.3 nA, and V_{mod} = 50 meV.

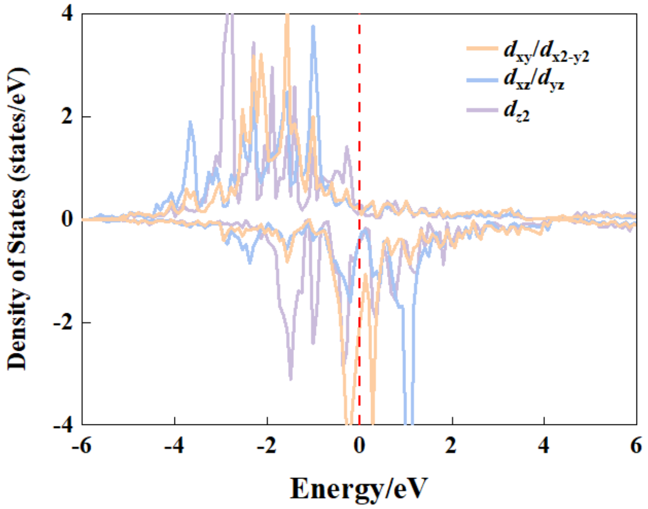

Figure S22. Electronic structure of FeSb films. Density of states contributed from Fe 3*d*-orbitals. The Fermi level (red dashed line) is set to zero.

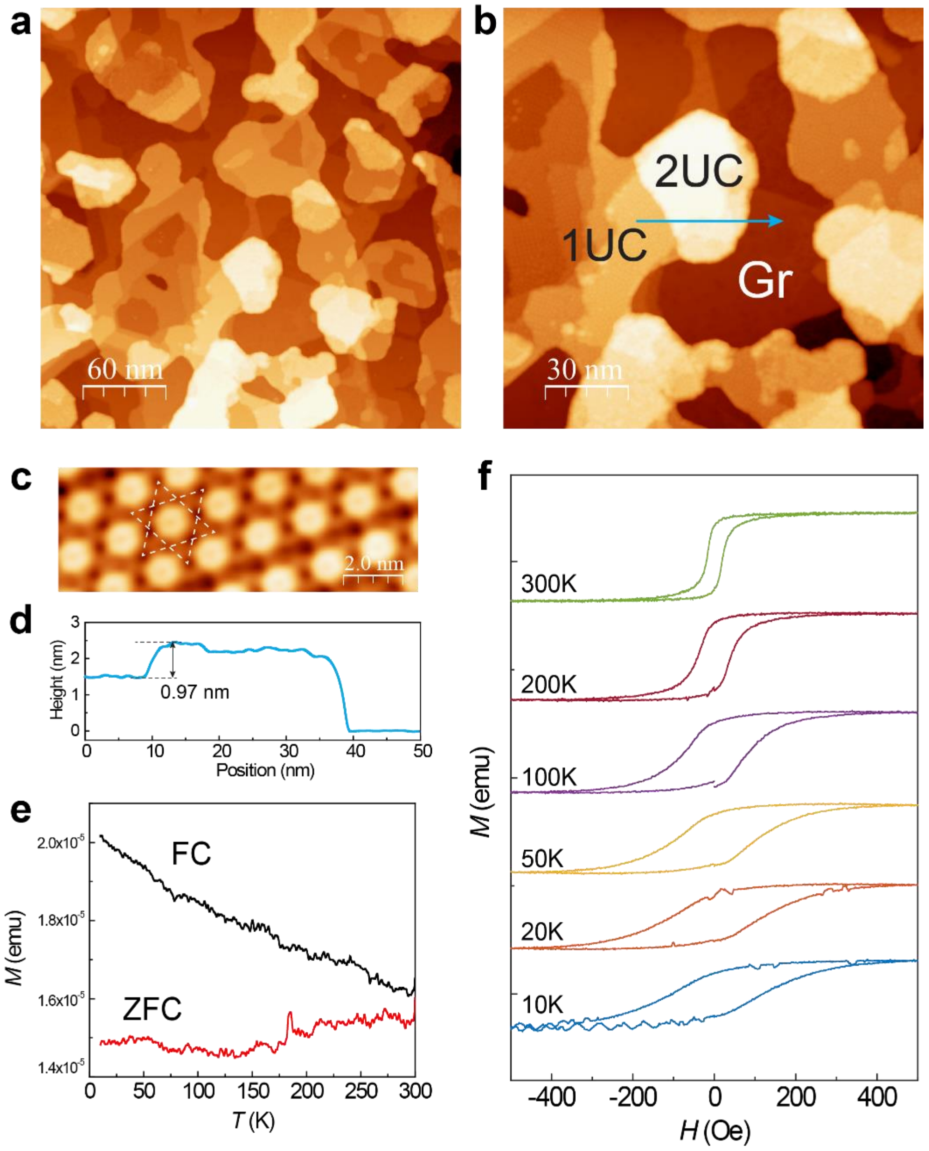

Figure S23. Reference sample: FeSb/STO films grown on bilayer graphene (Gr)/SiC(0001) substrate. (a) Topographic STM image of FeSb/bilayer Gr/SiC(0001) films. Setpoint: $V = 1.0$ V, $I = 1.0$ nA. (b) Zoom-in region showing 1BL, 2BL FeSb films, and the Gr substrate. Setpoint: $V = 1.0$ V, $I = 1.0$ nA. (c) STM image revealing the Kagome lattice, confirming the phase is similar to that grown on the STO(001) substrate. Setpoint: $V = 1.0$ V, $I = 1.0$ nA. (d) Line profile along the cyan arrow in (b). The height of the one-unit-cell film is 0.97 nm. (e) *M*-*T* curves of FeSb/bilayer Gr/SiC(0001) film. The FC is cooled in 1000 Oe and warmed up in 10 Oe, while ZFC is warmed up in 10 Oe. (f) *M*-*H* hysteresis loops measured at various temperatures up to 300 K.

Table S1 Phase information of Fe-Sb binary compositions. All the data comes from: https://materialsproject.org.

Composition	Crystal structure	Space group	Lattice parameters			Magnetic property	Magnetic moment
			a	***b***	***c***		
**Fe_3Sb**	$P6_3/mmc$	Hexagonal	5.494Å	5.494Å	4.360Å	FM	7.449μ_B
**Fe_4Sb_3**	$P\bar{6}m2$	Hexagonal	4.069Å	4.069Å	9.719Å	FM	7.931μ_B
**FeSb**	$P6_3/mmc$	Hexagonal	4.015Å	4.015Å	5.020Å	FM	1.273μ_B
**$FeSb_2$**	Pnnm	Orthorhombic	3.276Å	5.779Å	6.520Å	NM	0.853μ_B
**$FeSb_2$**	I_4/mcm	Tetragonal	5.763Å	5.310Å	5.310Å	FM	0.148μ_B
**$FeSb_3$**	$Im\bar{3}$	Cubic	7.949Å	7.949Å	7.949Å	FM	1.001μ_B

Table S2. The magnetic parameters for monolayer (ML) and bilayer (BL) FeSb films with Fe-termination and Sb-termination. The DFT calculated magnetic moment M_{Fe}^{PBE}, the evaluated magnetic moment by corresponding experiments data M_{Fe}^{Exp}, and magnetic anisotropy energy (MAE) per unit cell.

**Layer**	**ML**		**BL**	
	**Sb-ter**	**Fe-ter**	**Sb-ter**	**Fe-ter**
$\boldsymbol{M_{Fe}^{PBE}}$ (μ_B)	1.81	2.38	1.79	2.04
$\boldsymbol{M_{Fe}^{Exp}}$ (μ_B)	1.69	1.11	0.85	0.65
MAE (meV)	1.54	1.06	1.88	2.06

Table S3. The DFT calculated magnetic anisotropy energy (MAE) per Fe atom for both ultrathin Fe and bilayer FeSb films with Fe-termination and Sb-termination.

**Structure**		**MAE (meV)**	**Easy axis**
**Fe**	BCC Fe	0.031	out-of-plane
**BL**	Fe-ter	0.412	in-plane
	Sb-ter	0.469	in-plane

Table S4. Calculated electron occupation numbers in spin-up (O_{up}) and spin-down (O_{down}) states of Fe ions in bilayer FeSb with Fe-termination and Sb-termination. The values of O_{up}-O_{down} are 2.02, 1.90, 1.93 and 1.83 for $FeSb_x$ films, which are consistent with the values of magnetic moment from DFT calculations (M_{Fe}^{PBE}).

<table>
<tr><th colspan="3">Structure</th><th>d_{xy}</th><th>d_{yz}</th><th>d_{z2}</th><th>d_{xz}</th><th>d_{x2-y2}</th><th>d_{total}</th><th>O_{up}-O_{down}</th></tr>
<tr><td rowspan="5">BL</td><td rowspan="2">Fe-ter</td><td>O_{up}</td><td>0.84</td><td>0.88</td><td>0.86</td><td>0.88</td><td>0.84</td><td>4.30</td><td rowspan="2">2.02</td></tr>
<tr><td>O_{down}</td><td>0.52</td><td>0.34</td><td>0.56</td><td>0.34</td><td>0.52</td><td>2.28</td></tr>
<tr><td rowspan="3">Sb-ter</td><td>O_{up}</td><td>0.83</td><td>0.85</td><td>0.86</td><td>0.85</td><td>0.83</td><td>4.21</td><td rowspan="3">1.90</td></tr>
<tr><td>O_{down}</td><td>0.52</td><td>0.40</td><td>0.48</td><td>0.40</td><td>0.52</td><td>2.31</td></tr>
<tr><td>O_{down}</td><td>0.52</td><td>0.38</td><td>0.50</td><td>0.38</td><td>0.52</td><td>2.30</td></tr>
</table>

Table S5. The calculated Stoner parameters $D(E_F)$ (eV^{-1}) and I (eV) for bilayer FeSb films with Fe-termination and Sb-termination.

Layer	BL	
	Fe-ter	Sb-ter
$D(E_F)$	1.91	1.50
I	1.66	0.97
$D(E_F)\times I$	3.17	1.45

REFERENCES

(1) Yankovich, A. B.; Berkels, B.; Dahmen, W.; Binev, P.; Sanchez, S. I.; Bradley, S. A.; Li, A.; Szlufarska, I.; Voyles, P. M. Picometre-Precision Analysis of Scanning Transmission Electron Microscopy Images of Platinum Nanocatalysts. *Nat. Commun.* **2014**, *5* (1), 4155. https://doi.org/10.1038/ncomms5155.

(2) Liu, Z. Q.; Lü, W. M.; Lim, S. L.; Qiu, X. P.; Bao, N. N.; Motapothula, M.; Yi, J. B.; Yang, M.; Dhar, S.; Venkatesan, T.; Ariando. Reversible Room-Temperature Ferromagnetism in Nb-Doped SrTiO3 Single Crystals. *Phys. Rev. B* **2013**, *87* (22), 220405. https://doi.org/10.1103/PhysRevB.87.220405.

(3) Yu, Y.; Fu, H.; She, L.; Lu, S.; Guo, Q.; Li, H.; Meng, S.; Cao, G. Fe on Sb(111): Potential Two-Dimensional Ferromagnetic Superstructures. *ACS Nano* **2017**, *11* (2), 2143–2149. https://doi.org/10.1021/acsnano.6b08347.

(4) Yue, Y. Fe2C Monolayer: An Intrinsic Ferromagnetic MXene. *Journal of Magnetism and Magnetic Materials* **2017**, *434*, 164–168. https://doi.org/10.1016/j.jmmm.2017.03.058.

(5) Li, Z.-H.; Wang, J.; Yuan, J.-H. Prediction of Two-Dimensional M2As (M = Mn, Fe) with High Curie Temperature and Large Perpendicular Magnetic Anisotropy. *Computational Materials Science* **2021**, *200*, 110838. https://doi.org/10.1016/j.commatsci.2021.110838.

(6) Schneider, C. M.; Bressler, P.; Schuster, P.; Kirschner, J.; de Miguel, J. J.; Miranda, R. Curie Temperature of Ultrathin Films of Fcc-Cobalt Epitaxially Grown on Atomically Flat Cu(100) Surfaces. *Phys. Rev. Lett.* **1990**, *64* (9), 1059–1062. https://doi.org/10.1103/PhysRevLett.64.1059.

(7) Stampanoni, M.; Vaterlaus, A.; Aeschlimann, M.; Meier, F. Magnetism of Epitaxial Bcc Iron on Ag(001) Observed by Spin-Polarized Photoemission. *Phys. Rev. Lett.* **1987**, *59* (21), 2483–2485. https://doi.org/10.1103/PhysRevLett.59.2483.

(8) Steglich, F. Experimental Study of Ce-Based Heavy-Fermion Compounds. *Journal of Magnetism and Magnetic Materials* **1991**, *100* (1), 186–203. https://doi.org/10.1016/0304-8853(91)90820-Z.

(9) Zhang, T.; Spangenberg, M.; Greig, D.; Takahashi, N.; Shen, T.-H.; Matthew, J. a. D.; Cornelius, S.; Rendall, M.; Seddon, E. A. Ultrathin Epitaxial Fe Films on Vicinal GaAs(001): A Study by Spin-Resolved Photoelectron Spectroscopy. *Appl. Phys. Lett.* **2001**, *78* (7), 961–963. https://doi.org/10.1063/1.1345820.

(10) Kul'kova, S. E.; Eremeev, S. V.; Postnikov, A. V.; Bazhanov, D. I.; Potapkin, B. V. Atomic and Electron Structure of the GaAs (001) Surface. *Semiconductors* **2007**, *41* (7), 810–817. https://doi.org/10.1134/S106378260707007X.

(11) Xu, Y. B.; Kernohan, E. T. M.; Freeland, D. J.; Ercole, A.; Tselepi, M.; Bland, J. A. C. Evolution of the Ferromagnetic Phase of Ultrathin Fe Films Grown on GaAs(100)-4x6. *Phys. Rev. B* **1998**, *58* (2), 890–896. https://doi.org/10.1103/PhysRevB.58.890.

(12) Burch, K. S.; Mandrus, D.; Park, J.-G. Magnetism in Two-Dimensional van Der Waals Materials. *Nature* **2018**, *563* (7729), 47–52. https://doi.org/10.1038/s41586-018-0631-z.

(13) Zakeri, Kh.; Kebe, Th.; Lindner, J.; Farle, M. Power-Law Behavior of the Temperature Dependence of Magnetic Anisotropy of Uncapped Ultrathin Fe Films on GaAs(001). *Phys. Rev. B* **2006**, *73* (5), 052405. https://doi.org/10.1103/PhysRevB.73.052405.
